# Intermolecular Bending States and Tunneling Splittings of Water Trimer from Rigorous 9D Quantum Calculations: I. Methodology, Energy Levels, and Low-Frequency Spectrum

**DOI:** 10.1021/acs.jpca.4c05045

**Published:** 2024-09-16

**Authors:** Peter M. Felker, Irén Simkó, Zlatko Bačić

**Affiliations:** † Department of Chemistry and Biochemistry, University of California, Los Angeles, California 90095-1569, United States; ‡ Department of Chemistry, New York University, New York, New York 10003, United States; § Simons Center for Computational Physical Chemistry, New York University, New York, New York 10003, United States; ∥ NYU-ECNU Center for Computational Chemistry, NYU Shanghai, Shanghai 200062, China

## Abstract

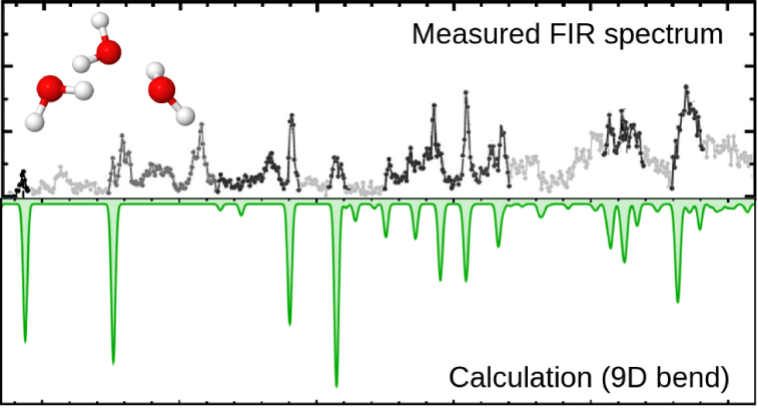

We present the computational methodology that enables the first rigorous nine-dimensional (9D) quantum calculations of the intermolecular bending states of the water trimer, as well as its low-frequency spectrum for direct comparison with experiment. The water monomers, treated as rigid, have their centers of mass (cm’s) at the corners of an equilateral triangle, and the intermonomer cm-to-cm distance is set to a value slightly larger than that in the equilibrium geometry of the trimer. The remaining nine strongly coupled large-amplitude bending (angular) degrees of freedom (DOFs) enter the 9D bend Hamiltonian of the three coupled 3D rigid-water hindered rotors. Its 9D eigenstates encompass excited librational vibrations of the trimer, as well as their torsional and bifurcation tunneling splittings, which have been the subject of much interest. The calculations of these eigenstates are extremely demanding, and a sophisticated computational scheme is developed that exploits the molecular symmetry group of the water trimer, *G*_48_, in order to make them feasible in a reasonable amount of time. The spectrum of the low-frequency vibrations of the water trimer simulated using the eigenstates of the 9D bend Hamiltonian agrees remarkably well with the experimentally observed far-infrared (FIR) spectrum of the trimer in helium nanodroplets over the entire frequency range of the measurements from 70 to 620 cm^–1^. This shows that most peaks in the experimental FIR spectrum are associated with the intermolecular bending vibrations of the trimer. Moreover, the ground-state torsional tunneling splittings from the present 9D calculations are in excellent agreement with the spectroscopic data. These results demonstrate the high quality of the *ab initio* 2 + 3-body PES employed for the DOFs included in the bound-state calculations.

## 1 Introduction

High-resolution microwave and far-infrared (FIR) spectra of small water clusters in molecular beams, in combination with high-level electronic structure and quantum dynamics treatments, can provide uniquely detailed information regarding the hydrogen-bond rearrangement dynamics (HBRD) and cooperativity effects in hydrogen bonding. These features are of central importance for molecular-level understanding of the structural and dynamical properties of liquid and solid phases of water and aqueous solutions.^1,2^

For a long time, the focus of both spectroscopic and theoretical studies was predominantly on the water dimer.^3−6^ However, more recently, increasing attention has been directed at the water trimer, undoubtedly the most important hydrogen-bonded trimer.^7−11^ It is the smallest water cluster in which nonadditive three-body interactions and cooperative hydrogen bonding can manifest, both of which play a major role in shaping the energetics, structural, and dynamical properties of liquid and solid phases of water, and their accurate description is therefore of fundamental importance.

The water trimer has the cyclic *C*_1_ equilibrium structure, in which each water monomer acts as a proton donor to one monomer and as a proton acceptor to the other.^7,11^ In this equilibrium structure, shown in Figure 2a, two free, or dangling, hydrogen atoms (not forming hydrogen bonds) are above the plane defined by the three oxygen atoms (up or *u*) and the third is below this plane (down or *d*). The apparent simplicity of this equilibrium structure, denoted as *uud*,^12^ is deceptive. The topology of the potential-energy surface (PES) of the trimer is in fact complex, owing to its high symmetry. There are 48 equivalent, isoenergetic minima identical to the equilibrium structure above, that are accessible without breaking any covalent bonds or the interconversion between the clockwise (cw) and counterclockwise (ccw) arrangements of the hydrogen bonds of the trimer. What gives rise to the surprisingly rich HBRD is the presence of the two low-barrier tunneling pathways linking the 48 equivalent potential minima.^13−16^ The one that has received by far the most attention involves the large-amplitude torsional or flipping motion of the free O–H bonds around the hydrogen bonds in the plane of the trimer. If only this torsional motion is feasible, the corresponding molecular-symmetry (MS) group is *G*_6_, isomorphic to the *C*_3*h*_ point group. Under *G*_6_, each rotation-vibration energy level of the trimer is predicted to split into four sublevels, two of which are nondegenerate and two doubly degenerate.^11,13^ The second tunneling pathway involves concerted breaking and reforming of the trimer hydrogen bonds. It is called donor tunneling, and it involves the interchange of the hydrogen-bonded and free H atoms of a water monomer. It is also referred to as bifurcation tunneling, since it proceeds through a transition state where both protons of a water monomer are donated to a hydrogen bond with a neighboring monomer.^14^ If feasible, the bifurcation pathway results in further splitting of each level in the *G*_6_ MS group into a quartet (*A*_1_^±^ levels) or sextet (*A*_2/3_^±^ levels), labeled by the irreducible representations of the MS group *G*_48_. Such tunneling splittings of rovibrational transitions have been observed in the FIR spectra of (H_2_O)_3_ and (D_2_O)_3_,^8^ establishing that *G*_48_ is indeed the appropriate MS group for such water trimers with no mixed isotopes.^8,11^ The measured torsional and bifurcation tunneling splittings differ greatly in magnitude;^11^ while the torsional manifold of the ground vibrational state extends to excitation energies up to 90 cm^–1^, the ground-state bifurcation tunneling splittings are very small, 40–300 MHz.

Torsional levels of (H_2_O)_3_ and isotopologues have attracted a great deal of attention from theorists, and have been calculated by a variety of approaches for the ground vibrational state of the trimer. One of them is the three-dimensional (3D) quantum treatment of the coupled large-amplitude torsional vibrations of the trimer, in which the only motions considered are those of the free O–H(D) bonds, constrained to rotate (in 1D) around the three hydrogen-bonded O–H(D) bonds. All other inter- and intramolecular vibrations of the trimer are taken to be frozen. This approach was introduced by Bačić, Leutwyler, and coworkers,^15,17,18^ [who extended this treatment to a (3 + 1)-dimensional model that took into account the coupling between the torsional motion and the symmetric H-bond stretch^19,20^] and also by van der Avoird and coworkers for the case of the rotating trimer.^16,21,22^ In addition, the torsional tunneling splittings have been calculated by the DMC methods,^23−25^ instanton theory,^26,27^ and PIMD,^28,29^ but only for the ground vibrational state of the trimer. Finally, an attempt was made to calculate the tunneling splittings in the six lowest-frequency intermolecular normal modes of the trimer using the modified WKB method.^30^ However, the normal-mode, harmonic description employed is not appropriate for these modes which in reality are highly anharmonic and exhibit large-amplitude motions. Therefore, the results cannot be expected to be quantitative and can at best indicate trends.

The water trimer has 21 vibrational modes, of which 9 are intramolecular and 12 are intermolecular. There are three types of the latter vibrational modes: torsional (described above), translational (also referred to as intermolecular stretching or H-bond stretching), and librational (coupled hindered rotations of the water monomers). Of course, these labels need to be taken with caution, as the different types of modes are coupled. Until rather recently, all FIR spectroscopic studies focused on the frequency region below 100 cm^–1^ dominated by the manifold of the torsional states.^11^ The only exceptions to this were the observations of the translational band of (D_2_O)_3_ at 142.8 cm^–1^ and four bands measured^11^ for (H_2_O)_3_ between 510 and 525 cm^–1^. With regard to the latter, three of the bands were assigned as bifurcation tunneling components of an excited librational mode.^31^ The implication of this assignment is that the excitation of this intermolecular mode increases dramatically the bifurcation tunneling splitting by several orders of magnitude, compared to the ground vibrational state.^11^

Our very limited view of the intermolecular vibrations of the water trimer was greatly expanded by the recent impressive study reporting the remarkable far-infrared (FIR) spectrum of (H_2_O)_3_ in helium nanodroplets.^25^ The recorded spectrum, discussed in more detail later, covers the low-frequency region from 70 to 620 cm^–1^ which includes all three types of the intermolecular vibrations of the trimer, torsional, intermolecular stretching, and librational vibrations. Therefore, a plethora of peaks visible in this FIR spectrum must correspond to the excitations of these intermolecular vibrations.

The water trimer is the obvious candidate for sensitive testing of the computed 3-body interactions through comparison of the intermolecular vibration–rotation-tunneling (VRT) states from high-level quantum bound-state calculations on a state-of-the-art 2 + 3-body potential energy surface (PES) of the trimer with the growing body of spectroscopic data. The newly available FIR spectrum of this trimer^25^ of unprecedented scope and level of detail provides an ideal opportunity for the comprehensive assessment of the accuracy of any available intermolecular PES (IPES) of the trimer and guiding its refinement.

However, this task is both formally and computationally highly demanding, and its realization requires methodology for computing rigorously the VRT states of water trimer that did not exist until now. The methods mentioned earlier used to calculate the torsional tunneling splittings of the trimer are not applicable to excited intermolecular vibrational states in general. In ref (25), the intermolecular vibrational states were calculated by means of the second-order vibrational perturbation theory (VPT2). The results of the VPT2 calculations were useful for assigning certain excited states, but in numerous instances only tentative assignments could be made or none at all. Especially challenging proved to be the description of the torsional manifolds in excited translational and librational modes, for which the VPT2 is largely inadequate. The general problem of calculating accurately the excited intermolecular vibrational states of the water trimer, particularly those exhibiting large-amplitude motions, together with their tunneling splittings remained unsolved. We cite the final paragraph of ref (25): “However, to describe the manifold of torsional states for the translational and librational modes accurately, a treatment of the water trimer in a 12-dimensional model (including all 12 intermolecular modes) is inevitable. This remains one of the challenges of future theoretical studies.”

In this paper we make a significant step toward meeting this challenge, which is considerable. A 21D quantum treatment of the coupled intra- and intermolecular vibrations of water trimer is not feasible at the present time (although this may change in the future). What is feasible now are 12D rigid-monomer fully coupled quantum calculations of the intermolecular VRT states of water trimer. We have already performed rigorous 12D quantum calculations of the coupled intra- and intermolecular vibrational states of HF trimer^32^ and HCl trimer.^33^ The methodology which enabled these calculations for the first time was developed by us recently.^32^ For the trimers of diatomic molecules, such calculations are full-dimensional. Thus, the dimensionality (12D) of the vibrational problem of the water trimer in the rigid-monomer approximation is something we have dealt with previously. The assumption of rigid monomers for computing intermolecular vibrational states of water trimer is reasonable in view of the large disparity between the intramolecular stretch and bend frequencies of the water monomers and the frequencies of the intermolecular vibrations. The 12D rigid-monomer calculations for the water trimer are expected to be considerably more difficult than those for the 9D rigid-monomer HX trimers (X = F, Cl)^34^ for two reasons. One is obviously the higher dimensionality of the former. The second is the elaborate hierarchy of torsional and bifurcation tunneling splittings of very different magnitudes that is superimposed on the VRT states of the water trimer. In contrast, no tunneling splittings have been observed, in the measured spectra and quantum calculations, of HX trimers (X = F, Cl).

The computational strategy planned for the rigorous 12D (rigid monomer) quantum calculations of the VRT states of the water trimer mirrors that employed in our 9D rigid-monomer quantum calculations of the intermolecular vibrational states of the HF trimer.^34^ The full 12D rigid-monomer VRT Hamiltonian of the water trimer is partitioned into a 3D frame (intermolecular stretching) Hamiltonian and a 9D bend Hamiltonian for three fully coupled 3D rigid-monomer rotors with the intermonomer center-of-mass-to-center-of-mass (cm-to-cm) distances fixed. The 9D bending eigenstates of the latter encompass excited librational vibrations with the torsional and bifurcation tunneling splittings superimposed on them. Each of these reduced-dimension Hamiltonians is diagonalized separately, the 9D bend Hamiltonian in the basis consisting of trilinear products of monomer hindered-rotor states. A certain number of their respective lowest-energy 3D and 9D eigenstates is included in the final 12D product contracted basis in which the full 12D intermolecular vibrational Hamiltonian (bend + frame) of the water trimer is diagonalized.

It can be mentioned here that solving for the intermolecular bending eigenstates of the 9D bend Hamiltonian of the water trimer is analogous to the earlier 6D quantum calculations of bending energy levels of the HF trimer by Wang and Carrington,^35^ also performed for rigid monomers (diatomic in this case) and fixed intermonomer distances. But the results of the latter calculations were not intended to be a component of a higher-dimensional treatment of the HF trimer vibrations, in contrast to the present eigenstate calculations for the 9D bend Hamiltonian.

In this work, our focus is on solving the 9D bend problem of the water trimer. The resulting 9D intermolecular eigenstates naturally include those arising from the torsional and bifurcation tunneling splittings in excited intermolecular states of the trimer. This constitutes the most rigorous, high-dimensional quantum treatment to date of the excited intermolecular vibrational states of the water trimer and their tunneling splittings, even without the inclusion of the intermolecular stretching degrees of freedom (DOFs). The *ab initio* 2 + 3-body PES of the water trimer by Zhang et al.^29^ is used in the present 9D calculations. Full-dimensional PIMD calculations on this PES gave the energies of the levels in the lowest torsional manifold of the trimer in excellent agreement with experimental values.^29^

The dimensionality of the bend Hamiltonian, 9D, is much higher than that of the 3D frame Hamiltonian, making the determination of its eigenstates much more demanding. Moreover, the nine DOFs of the bend problem are all large-amplitude and strongly coupled. In addition, a very large number of 9D potential-energy matrix elements need to be calculated for the PES that consists of not only two-body water–water terms but significant three-body terms as well. Finally, the 9D product contracted basis in which the 9D Hamiltonian is diagonalized must be made as efficient and compact as possible, in order to make the calculations feasible and reasonably fast on multiple processors. To accomplish all this, an elaborate scheme is devised, which fully exploits the *G*_48_ symmetry of the trimer, and its comprehensive description later in the paper is lengthy.

It is worth restating that computing the 9D bend eigenstates provides the key constituents of the 12D basis for the rigorous full 12D (stretch + bend) quantum calculations of the intermolecular vibrational states of the water trimer, to be reported in the near future.

The initial implementation of the newly developed methodology made in this work reveals that solving the 9D bend problem, besides providing the essential component of the final 12D intermolecular basis, already yields a rather accurate and comprehensive description of the intermolecular bending states and tunneling of the trimer. The calculated energies of the levels in the lowest torsional manifold agree very well with the experimental values. In addition, there is a remarkably good agreement between the measured FIR spectrum^25^ and the low-frequency spectrum of the trimer simulated using the 9D eigenstates in this work. This demonstrates that many of the most intense transitions in the experimental FIR spectrum are associated with the excitation of the primarily bending vibrations in the 9D subspace. Taken together, this implies that (a) the 9D bend Hamiltonian is physically meaningful in its own right, as it accounts for many of the most important features of the intermolecular VRT dynamics of the trimer including the tunneling splittings in the excited states, (b) the coupling between the bending and intermolecular stretching vibrations (the latter frozen in the 9D calculations) is likely to be rather weak, and (c) the PES^29^ utilized in these calculations is very accurate, certainly for the monomers in their ground state.

The 9D calculations carried out in this work set the stage for the rigorous 12D (rigid-monomer) quantum calculations of the VRT states of the water trimer with full coupling of the intermolecular stretching and bending DOFs. These calculations are well under way in our group and the results will be reported in the near future.

The computational scheme employed in calculating the 9D bending eigenstates of the water trimer is detailed in Section 2. Section 3 presents and discusses the results of the 9D calculations: the resulting low-energy bending states together with the torsional and bifurcation tunneling splittings. The low-frequency spectrum of the water trimer simulated using the 9D eigenstates is also presented in this section and compared to the measured FIR spectrum of water trimer in He nanodroplets. Section 4 contains the conclusions and the directions of future work on this topic.

## 2 Computational Methodology

### 2.1 Overview

**Figure 1:**
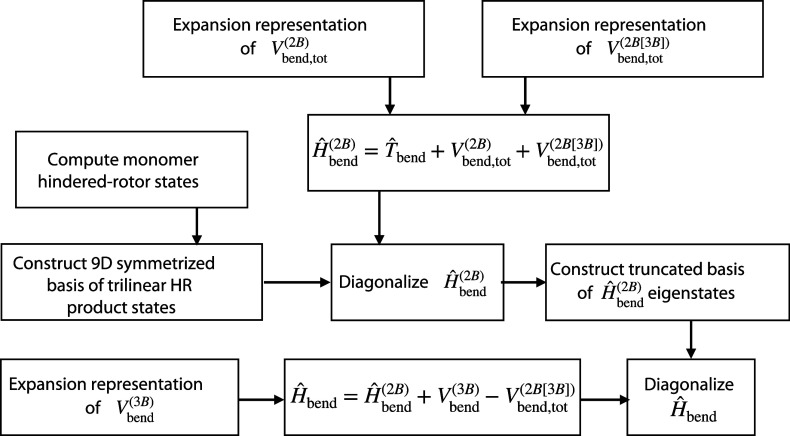
Flowchart depicting the major steps involved in diagonalizing the water-trimer bend Hamiltonian, *Ĥ*_bend_. In the figure *Ĥ*_bend_^(2B)^ is a two-body portion of *Ĥ*_bend_. *T̂*_bend_ is the bend kinetic-energy operator. *V*_bend,tot_^(2B)^ is the full two-body part of the bend potential-energy surface (PES). *V*_bend,tot_^(2B[3B])^ is the full two-body approximation to the three-body part of the bend PES, *V*_bend_^(3B)^. “HR” abbreviates “hindered-rotor”.

There are two particular challenges to surmount in computing the intermolecular bending eigenvectors of water trimer via a variational approach. First, the problem involves nine highly coupled large-amplitude intermolecular bending DOFs, with the rigid-monomer geometries and the intermonomer cm-to-cm distances set to the fixed values defined in Section 2.10. The 9D primitive basis employed to solve the problem must efficiently cover this space, suggesting, ostensibly, the need for something on the order of 10^9^ such functions. Second, the IPES^29^ consists of not just water–water (two-body - *V*_bend_^(2B)^) terms, but also significant water–water–water (three-body - *V*_bend_^(3B)^) terms. A grid representation of *V*_bend_^(3B)^ suitable for the calculation of matrix elements in the 9D primitive basis requires of order 2 × 10^10^ grid points (with full exploitation of symmetry). In the calculation of such matrix elements, not only must *V*_bend_^(3B)^ be computed for each grid point, but all of the 9D basis functions must be expressed on the grid, as well. These challenges are of such a magnitude that we have paid particular attention to trying to mitigate them. In so doing, we have settled on a scheme involving the following elements, which scheme is summarized visually in the flowchart of Figure 1.

First, we employ a primitive basis consisting of trilinear products of monomer hindered-rotor states. The 3D hindered-rotor states correspond to the rotational eigenstates of a given monomer in the force-field of two near-by, geometrically fixed water moieties in an arrangement approximating the equilibrium geometry of water trimer. In this way we build much of the dynamics of the water monomers within the trimer into the primitive basis. In producing these hindered-rotor states we pay close attention to symmetry so that ultimately the 9D trilinear hindered-rotor basis functions have well-defined transformation properties with respect to the operations of *G*_48_, the water-trimer molecular symmetry group.

Second, we employ this primitive basis to solve that large portion of the bend problem that consists solely of one-body and two-body terms. The corresponding Hamiltonian, *Ĥ*_bend_^(2B)^, is readily diagonalized into blocks corresponding to the irreducible representations (irreps) and subirreps (in the case of the 3D irreps) of *G*_48_. These blocks can be straightforwardly diagonalized individually by an iterative eigensolver (we use the Chebyshev version^36^ of filter diagonalization^37^), as the relevant hindered-rotor basis-set sizes required to achieve acceptable convergence are on the order of 10^5^ to 10^6^ functions per irrep/subirrep.

Third, we include in *Ĥ*_bend_^(2B)^ not only the pairwise sum over the two-body water–water bend potential for the trimer, but also a sum-over-two-body approximation of the three-body portion of the trimer’s bend PES, which we call *V*_bend,tot_^(2B[3B])^. This facilitates the final step in the diagonalization of the bend Hamiltonian.

Fourth, in that final step we diagonalize the full bend Hamiltonian, *Ĥ*_bend_, in a truncated basis of the eigenvectors obtained by diagonalizing *Ĥ*_bend_^(2B)^. The only nonzero off-diagonal matrix elements in this calculation correspond to those involving the difference between the three-body PES and our two-body approximation to that PES – i.e., [*V*_bend_^(3B)^ – *V*_bend,tot_^(2B[3B])^]. The magnitudes of these matrix elements are such that reasonable convergence is attainable with basis sets consisting of 10^2^ to 10^3^ states per irrep/subirrep.

Finally, in order to circumvent the problems associated with computing the matrix elements of *V*_bend_^(3B)^ by quadrature over a large grid, we represent that function instead as an expansion over trilinear products of Wigner matrix elements. The number of expansion coefficients required to represent *V*_bend_^(3B)^ accurately is orders of magnitude less than the number of values of that function required for a grid representation sufficiently large to permit accurate calculation of matrix elements. Further, the angle integrals involved in matrix element calculations have analytical solutions when the expansion representation of *V*_bend_^(3B)^ is used.

This scheme, with extensive exploitation of symmetry, allows for the calculation of low-energy bend eigenstates of the trimer in reasonable time (a few weeks) with a multiprocessor (ca. 60 processors) computer. Moreover, the methodology is readily adapted to the problem of computing the full 12D intermolecular states (bend + frame) of the trimer, which we will report on in the near future.

### 2.2 Coordinates and Hamiltonian

**Figure 2:**
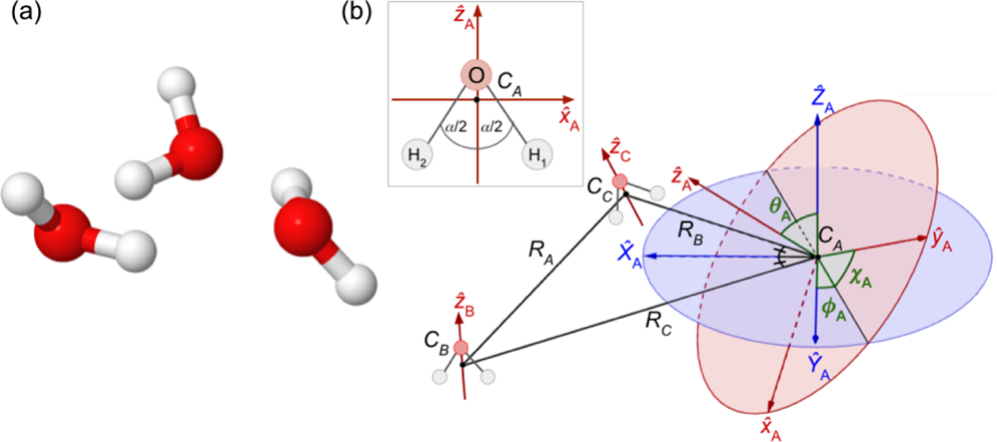
(a) Equilibrium structure of water trimer, corresponding to the *uud* arrangement. (b) The coordinates employed for computing the 9D intermolecular bending states of the water trimer. See text for the definitions. The intermonomer cm-to-cm distances *R*_*I*_ (*I* = *A*, *B*, *C*) are fixed and the water monomers are taken to be rigid.

The rigid-monomer vibrational Hamiltonian for water trimer can be obtained by generalization from the work of Wang and Carrington^38^ on the HF trimer. In that work the coordinates employed consist of (a) the three distances between monomer centers of mass (cm’s, denoted with *C*_*I*_, *I* = *A*, *B*, *C*) and (b) the angles that define the orientations of the monomer moieties with respect to “local” axis systems embedded in the trimer frame. To define analogous coordinates here [see Figure 2b], we label the three monomers *A*, *B*, and *C* and start with the position vectors of the monomer cm’s measured with respect to an arbitrary origin: **r**_*A*_, **r**_*B*_, and **r**_*C*_. We then define the inter-cm vectors as
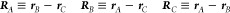
1The magnitudes of these vectors, *R*_*I*_ (*I* = *A*, *B*, *C*), constitute the “frame coordinates”. These inter-cm vectors are then also used to define the local Cartesian axes (*X̂*_*I*_, *Ŷ*_*I*_, *Ẑ*_*I*_) centered at the cm of each monomer. First, each *Ẑ*_*I*_ axis is defined to be parallel to **R**_*A*_ × **R**_*B*_ (i.e., normal to the plane formed by the monomer cm’s). Second, each *X̂*_*I*_ axis is defined to be parallel to the bisector of the interior angle of the triangle formed by the monomer cm’s with vertex at the cm of *I*. So, with *R̂*_*I*_ ≡ **R**_*I*_/*R*_*I*_,
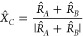
2
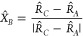
3
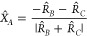
4Finally, *Ŷ*_*I*_ = *Ẑ*_*I*_ × *X̂*_*I*_. With the local axes defined, the local-angle bend coordinates, ω_*I*_ ≡ (ϕ_*I*_, θ_*I*_, χ_*I*_), are then defined as those Euler angles required to rotate the local axes (*X̂*_*I*_, *Ŷ*_*I*_, *Ẑ*_*I*_) into the monomer-fixed axes (*x̂*_*I*_, *ŷ*_*I*_, *ẑ*_*I*_) via the three-step transformation defined, for example, in ref (39), pp. 77–79. The monomer-fixed Cartesian axis systems (each centered at the cm of the relevant monomer, *C*_*I*_) are, in turn, defined by reference to the vectors from the monomer cm to the two H nuclei of the monomer:  and . In particular,  is taken to be antiparallel to the bisector of the acute angle formed by  and 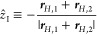
5 is taken to be normal to the monomer’s plane
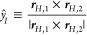
6and .

With the coordinates defined, one can write the rigid-monomer vibrational Hamiltonian of the water trimer as
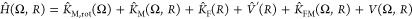
7where Ω denotes collectively (ω_*A*_, ω_*B*_, ω_*C*_) and *R* denotes collectively (*R*_*A*_, *R*_*B*_, *R*_*C*_). Expressions for the kinetic-energy terms , , , and  for water trimer are identical in form to those for HF trimer (see ref (38) and also eqs 3–9 of ref (34) and Section S1). The monomer rotational kinetic-energy operator  changes, however, in going from HF trimer to water trimer. For the latter, one has
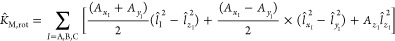
8where , ,  are the rigid-rotor monomer rotational constants associated with the principal axes , , and , respectively,  is the operator associated with the square of the rotational angular momentum of monomer *I*, and , , and  are the operators associated with the projection of the rotational angular momentum of monomer *I* along the , , and  axes, respectively.

Finally,  is the 12D, rigid-monomer potential-energy surface (PES), where the intramonomer coordinates are fixed to values given in Section 2.10. The PES we work with here (from Zhang et al.^29^) consists of sums over monomer–monomer two-body terms and a three-body term
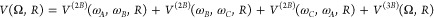
9Note that implicit in eq 9, and also in eq 7, are fixed values for the geometrical parameters that define the rigid-body monomer moieties.

In this work, we focus on the 9D water-trimer bend problem rather than the full 12D intermolecular (frame plus bend) problem associated with the Hamiltonian of eq 7. To obtain the relevant bend Hamiltonian we drop those terms in  that depend only on *R* and set  in the remaining terms (see Section 2.10 for ). This ultimately produces the following bend Hamiltonian for the case of identical monomers that we treat here
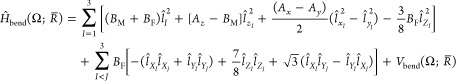
10where *B*_M_ ≡ (*A*_*x*_ + *A*_*y*_)/2 and *B*_F_ ≡ 1/(*MR̅*^2^) with *M* the mass of each monomer. Note that two different types of angular momentum operators enter into this expression–those that correspond to projections of rotational angular momentum along the monomer-fixed axes (*l̂* subscripts *x*_*I*_, *y*_*I*_, and *z*_*I*_) and those that correspond to projections of rotational angular momentum along the trimer local axes (*l̂* subscripts *X*_*I*_, *Y*_*I*_, and *Z*_*I*_). Finally, the 9D bend PE function, *V*_bend_, is simply defined as the function obtained when *V*(Ω, *R*) is evaluated at *R*_I_ = *R̅* for all *I*. As is the case for the 12D PE function, the bend PE function is composed of a sum over three two-body terms and a three-body term
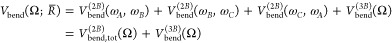
11where *V*_bend_^(2B)^(ω_*I*_, ω_*J*_) ≡ *V*^(2B)^(ω_*I*_, ω_*J*_; *R̅*),
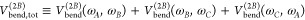
12and *V*_bend_^(3B)^(Ω) ≡ *V*^(3B)^(Ω; *R̅*).

### 2.3 Symmetry of *Ĥ*_bend_

The molecular symmetry group of feasible permutations and inversions of (H_2_O)_3_ and of (D_2_O)_3_ is well-known (see, for example, ref (11) to be *G*_48_ = {*E*, E*} ⊗ *G*_24_,^40^ where *E* is the identity operation and E* is the operator that inverts all nuclear and electronic coordinates. Above is a character table for *G*_24_ (Table 1), which is isomorphic with the point group *T*_*h*_:

**Table 1:** Character Table for *G*_24_

*G*_24_	1*E*	4(*ABC*)(135)(246)	4(*ACB*)(153)(264)	3 (12)(34)	1 (12)(34)(56)	4(*ABC*)(145236)	4(*ACB*)(163254)	3 (12)
*A*_1*g*_	1	1	1	1	1	1	1	1
*A*_2*g*_	1	ϵ^a^	ϵ*	1	1	ϵ	ϵ*	1
*A*_3*g*_	1	ϵ*	ϵ	1	1	ϵ*	ϵ	1
*T*_g_	3	0	0	–1	3	0	0	–1
*A*_1*u*_	1	1	1	1	–1	–1	–1	–1
*A*_2*u*_	1	ϵ	ϵ*	1	–1	–ϵ	–ϵ*	–1
*A*_3*u*_	1	ϵ*	ϵ	1	–1	–ϵ*	–ϵ	–1
*T*_*u*_	3	0	0	–1	–3	0	0	1

a ϵ ≡ e^*i*2π/3^.

The permutation operations of this group are denoted in the usual way with *A*, *B*, and *C* representing the O nuclei of monomers *A*, *B*, and *C*, respectively, and {1, 2}, {3, 4}, and {5, 6} representing {H nucleus #1, H nucleus #2} of monomers *A*, *B*, and *C*, respectively. The operations, irreducible representations (“irreps”), and character table of *G*_48_ are easily obtained from those of *G*_24_. The irreps of the former are denoted in the same way as those of *G*_24_ except with an additional right superscript of plus or minus signifying even or odd parity (i.e., transformation behavior with respect to E*), respectively.

It is straightforward to show that  of eq 10 is invariant with respect to the operations of *G*_48_. As such, each eigenvector of  must transform as one of the irreps of that group. Moreover, given a basis set consisting of functions that transform as *G*_48_ irreps, the matrix of  in that basis can be block-diagonalized into blocks corresponding to each irrep. We make use of this fact here in computing the eigenvectors of .

### 2.4 9D Basis States as Trilinear Products of Hindered-Rotor Eigenstates

We construct the 9D functions of the basis in which the matrix of  is expressed, and ultimately diagonalized, as products of 3D, hindered-rotor functions:
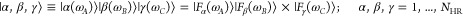
13Note that the order of α, β, γ in denoting these states has meaning. The first symbol refers to a hindered-rotor state dependent on ω_*A*_, the second to one dependent on ω_*B*_ and the third to one dependent on ω_*C*_.

The hindered-rotor functions, , etc., are computed in the following way. First, the hindered-rotor, 3D eigenvalue equation
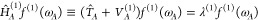
14is solved. Here,
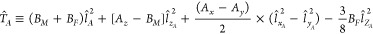
15is that part of the kinetic-energy portion of  that depends exclusively on ω_*A*_, the local angles associated with monomer A. *V*_*A*_^(1)^(ω_*A*_) is a 3D, symmetrized potential-energy function obtained from *V*_bend_:
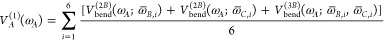
16where ω̅_*B*,*i*_, ω̅_*C*,*i*_ are the (fixed) Euler angles of monomers *B* and *C* that correspond to *i*-th version of the six equivalent trimer geometries obtained by repeated operation of (*ACB*)(153)(264)* on one of the 96 trimer geometries corresponding to the minimum of *V*_bend_. (The set of six ω̅_*B*,*i*_, ω̅_*C*,*i*_ that we choose corresponds to geometries in which hydrogen #4 is the donor in the B–A hydrogen bond and hydrogen #6 is the donor in the C–B hydrogen bond.) The operator *Ĥ*_A_^(1)^ is invariant to E* and to the permutation (12). As such, the eigenvectors of eq 14 are also eigenvectors of these two operators.

Second, a new 3D eigenvalue equation is constructed and solved by making use of the lowest-energy eigenvector [denoted *f*_1_^(1)^(ω)] from eq 14:
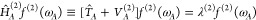
17where *V*_*A*_^(2)^(ω_*A*_) is the potential felt by monomer *A* when monomers *B* and *C* are in the hindered-rotor states corresponding to wave functions *f*_1_^(1)^(ω_*B*_) and *f*_1_^(1)^(ω_*C*_), respectively:
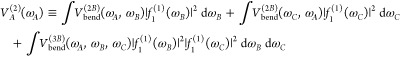
18The ground state of *Ĥ*_*A*_^(2)^ is then used to construct a new potential and new hindered rotor eigenvalue equation in the same manner in which the ground state of *Ĥ*_A_^(1)^ was used to construct eq 17. This new equation is solved, and the process is repeated until the set of λ^(*n*)^ eigenvalues matches (to within a cm^–1^ or so) the λ^(*n*–1)^ set. The resulting set of the *N*_HR_ lowest-energy hindered-rotor eigenfunctions
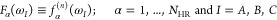
19is then taken as that with which to construct the functions of eq 13.

We computed the hindered-rotor states variationally (see Section S2 for further details) by using a basis of normalized symmetric-top rotational eigenstates
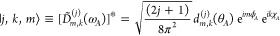
20where *j* = 0, ..., *j*_max_; *m*, *k* = −*j*, −*j* + 1, ..., *j*, and *d*_*m*,*k*_^(*j*)^ are “little-*d*” Wigner matrix elements (e.g., see eq 3.57 of ref (39)). The computed states are thus given as expansions over this basis:
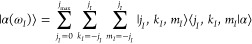
21

#### 2.4.1 Symmetry Considerations in Respect to the Hindered-Rotor States

Given that *f*_1_^(1)^(ω_*A*_) is an eigenvector of E*, it follows that both |*f*_1_^(1)^(ω_*B*_)|^2^ and |*f*_1_^(1)^(ω_*C*_)|^2^ are invariant with respect to E*. Both, too, are invariant with respect to the H-exchange operator associated with monomer A, i.e., (12). Thus, given the invariance of *V*_bend_^(2B)^(ω_*A*_, ω_*B*_) and *V*_bend_^(2B)^(ω_*C*_, ω_*A*_) with respect to these operators, it is clear that *V*_*A*_^(2)^(ω_*A*_), and, therefore, *Ĥ*_*A*_^(2)^, are invariant with respect E* and (12). Hence, all the *Ĥ*_*A*_^(2)^ eigenvectors, *f*_*j*_^(2)^(ω_*A*_), are also eigenvectors of E* and (12). It is easy to see that these symmetries carry through each step of the iterative process leading to the set of hindered-rotor states ultimately used to construct the bend basis. The upshot is that each hindered-rotor state |α(ω_*I*_)⟩ that composes the 9D basis is an eigenvector of E* with eigenvalue *p*_α_ = ±1 and also an eigenvector of the H-exchange operator associated with monomer *I* with eigenvalue *q*_α_ = ±1:
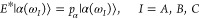
22and
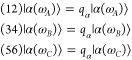
23

### 2.5 Symmetries of the 9D Primitive Basis States

The set of |α, β, γ⟩ primitive basis functions have well-defined transformation properties with respect to all of the operations of *G*_48_. We consider those properties below. First, however, it is important to point out that these basis functions are suitable only for calculations pertaining to one of the two enantiomeric forms of the trimer–the two forms distinguished by the way in which the H-bond donors and acceptors are arranged. In particular, by virtue of the way in which we compute the hindered-rotor states (specifically, tracing back to the way in which we define ), the basis states, and our calculations, apply only to the enantiomer in which monomer A is the proton donor to monomer C, monomer C is the proton donor to monomer B, and monomer B is the proton donor to monomer A. In the context of group theory, the set of basis states we use does not have well-defined transformation properties with respect to half of the operations of *G*_96_, the largest molecular symmetry group possible for the trimer without considering the breaking of covalent bonds. Given that there is no evidence that the tunneling between enantiomeric forms of the trimer produces observable splittings, this limitation is not a meaningful drawback. Indeed, it is an advantage, in that a *G*_96_ basis would certainly have to be larger to achieve the same level of convergence that characterizes a *G*_48_ basis of a given size.

Given eqs 22 and 23, the transformation of the |α, β, γ⟩ by all of the operations of *G*_48_ that involve just E* and/or any of the H-exchange operators can be trivially determined, as all of the basis states are eigenvectors of such operators. Thus, for example,
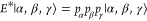
24and
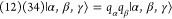
25

To determine the effect of the operations involving the permutation of monomers on these states, we start with a consideration of one such operator: (*ABC*)(135)(246), which moves monomer B to monomer-A’s position, monomer C to monomer-B’s position, and monomer A to monomer-C’s position, all such that monomer B’s new Euler angles  monomer A’s new Euler angles , and monomer C’s new Euler angles . The effect of the operator on |α, β, γ⟩ is thus given by^40^
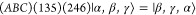
26The effect of all the other operators in the same class as (*ABC*)(135)(246) can then be easily determined since these other operators are each equivalent to the product of two H-exchange operators with (*ABC*)(135)(246). For example, (*ABC*)(235)(146) = (12)(34) × (*ABC*)(135)(246). Thus,
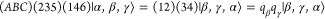
27Similarly, the effect of the all the operators in the (*ACB*)(153)(246) class can be determined by starting with
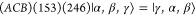
28and noting that all the other operations in the same class are each equivalent to the product of two H-exchange operators with (*ACB*)(153)(246). Finally, the operators in the (*ABC*)(145236) class are equivalent to the product of either one or three H-exchange operators with (*ABC*)(135)(246), and the operators of the (*ACB*)(163254) class are equivalent to the product of either one or three H-exchange operators with (*ACB*)(153)(264). Hence, the transformations by these of operators of |α, β, γ⟩ are also readily determined. As one example,
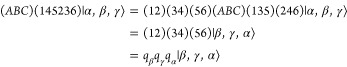
29In this way, and together with the fact that for any operator *Ô*, the operator Ô* = E*Ô, one can determine the transformation properties of all the primitive 9D basis functions with respect to all of the operations of *G*_48_.

With their transformation properties in hand, one can construct from the primitive basis states *G*_48_-symmetry-adapted basis states by well-known methods. Table 2 summarizes the results. Note that primitive basis states with only certain combinations of *q*_α_, *q*_β_, *q*_γ_ can contribute to symmetry-adapted states belonging to a particular irrep. Note also that primitive basis states for which *p*_α_*p*_β_*p*_γ_ = +1 only contribute to even-parity irreps and those for which *p*_α_*p*_β_*p*_γ_ = −1 only contribute to odd-parity irreps. Finally, note from the Table that we have chosen particular representations for the *T* irreps. We will henceforth refer to *T* states that transform in the same way as the basis states designated by the subscripts *a*, *b*, and *c* in Table 2 as belonging to *a*, *b*, or *c* “sub-irreps” of the *T* irrep in question.

**Table 2:** Characteristics of the Symmetry-Adapted Bend Basis States

Γ^a^	{*q*_α_, *q*_β_, *q*_γ_}	Symmetry-adapted basis-ket types^b^	# of Symmetry-adapted states^c^	# of Primitive states
*A*_1*g*/1*u*_	{±1, ±1, ±1}	|α, α, α⟩ and	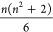	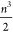
*A*_2*g*/2*u*_	{±1, ±1, ±1}	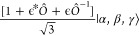	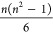	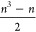
*A*_3*g*/3*u*_	{±1, ±1, ±1}	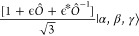	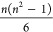	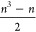
*T*_*g*,*a*_	{+1, −1, −1}	|α, β, γ⟩	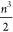	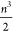
*T*_*g*,*b*_	{−1, +1, −1}	|α, β, γ⟩	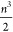	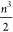
*T*_*g*,*c*_	{−1, −1, +1}	|α, β, γ⟩	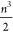	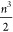
*T*_*u*,*a*_	{−1, +1, +1}	|α, β, γ⟩	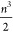	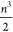
*T*_u,b_	{+1 ,–1, +1}	|α, β, γ⟩	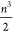	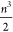
*T*_*u*,*c*_	{+1, +1, −1}	|α, β, γ⟩	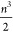	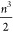

a States belonging to Γ^+^ irreps have *p*_α_*p*_β_*p*_γ_ = +1. States belonging to Γ^–^ irreps have *p*_α_*p*_β_*p*_γ_ = −1.

b ϵ ≡ e^*i*2π/3^ and *Ô* ≡ (*ABC*)(135)(246).

c Under the assumption that there are a total of *N*_HR_/4 each of the four symmetry types of hindered-rotor states. See the text. *n* ≡ *N*_HR_/2.

In the fourth column of Table 2 we list the sizes of the symmetry-specific basis sets under the assumption that, of the total number of hindered-rotor states, *N*_HR_, one-quarter each correspond to (*p*_α_, *q*_α_) = (+1, +1), (+1, −1), (−1, +1), and (−1, −1), respectively. (Clearly, this assumes that *N*_HR_ is a multiple of 4.) The sizes pertain to, and are equal for, both the even- and odd-parity irreps associated with a given row in the Table. In the far-right column of the Table we list the total number of primitive basis functions for each irrep/subirrep under the same assumption.

### 2.6 Solving for the Eigenstates of *Ĥ*_bend_: General Scheme

The largest impediment to diagonalizing *Ĥ*_bend_ in the bases we have detailed above arises from the need to deal with the not-insignificant *V*_bend_^(3B)^ part of that operator. The required basis-set sizes (of order 10^5^–10^6^ states) are large enough to mandate an iterative diagonalization scheme (e.g., filter diagonalization or Lanczos). In such schemes, one needs to evaluate the effect of the PE part of the Hamiltonian on a state vector expressed in the basis-set representation. This is typically handled by transforming that vector to a grid representation, multiplying that version of the vector by the PE function’s value at each grid point, and transforming the result back to the basis-set representation. This is done on order of hundreds or thousands of times. The problem is that the size of the 9D grid required to accurately represent *V*^(3B)^ and the state function is so large (of order 10^10^ points or larger) as to render this scheme unfeasible.

As a way to circumvent this problem, we have implemented a two-step approach to the diagonalization of *Ĥ*_bend_. In the first step we diagonalize, in the basis described in the preceding subsection, the operator
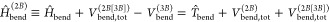
30where *T̂*_bend_ is the kinetic-energy portion of *Ĥ*_bend_, and *V*_bend,tot_^(2B[3B])^ is a sum over **two-body approximation** terms to *V*_bend_^(3B)^:
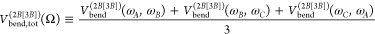
31where the *V*_bend_^(2B[3B])^(ω_*I*_,ω_*J*_) are given by
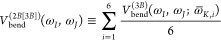
32and the six sets of fixed angles  (*i* = 1–6) correspond to the symmetrically equivalent equilibrium values of ω_*K*_ connected by repeated application of the pseudorotation operator (*ABC*)(135)(246)*—see, for example, Figure 2a of ref (11). (We include *V*_bend,tot_^(2B[3B])^ in *Ĥ*_bend_^(2B)^ to improve convergence in the final step in the diagonalization of the full *Ĥ*_bend_—see below.) The diagonalization of *Ĥ*_bend_^(2B)^ is considerably less demanding than the diagonalization of the full *Ĥ*_bend_; the potential matrix elements are only 6D integrals and many of them have identical values. The upshot is that this first computation can be readily handled by an iterative eigensolver.

In the second step, we diagonalize the full
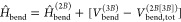
33in a truncated basis comprised of the lowest-energy eigenvectors of *Ĥ*_bend_^(2B)^. In this second step the only nontrivial, and the only off-diagonal, matrix elements that need to be computed are those of *V*_bend_^(3B)^ – *V*_bend,tot_^(2B[3B])^. One sees now the advantage of including *V*_bend,tot_^(2B[3B])^ in *Ĥ*_bend_^(2B)^: In so doing one reduces (significantly, it turns out) the overall magnitude of the off-diagonal matrix elements of *Ĥ*_bend_ in the *Ĥ*_bend_^(2B)^-eigenvector basis compared to the matrix elements that would obtain if it were not included. As a result, the size of the *Ĥ*_bend_^(2B)^-eigenvector bases required to achieve acceptable convergence in this last step is only of order several hundreds of states per *G*_48_ irrep/subirrep.

### 2.7 Diagonalization of *Ĥ*_bend_^(2B)^

We diagonalize the matrix of *Ĥ*_bend_^(2B)^ in the basis of eq 13 by the Chebyshev version of filter diagonalization (CFD).^36^ In so doing we make use of the fact that *Ĥ*_bend_^(2B)^ can be block diagonalized into 24 blocks (see Section S3) corresponding to the different sets of states labeled by irrep and subirrep and enumerated in Table 2. To exploit the ability to block-diagonalize within the CFD algorithm we arrange it so that the initial, random state vector, |Ψ_Γ_⟩, required to start the procedure belongs to a specific *G*_48_ irrep or subirrep, Γ, because the CFD algorithm applied to such an initial state vector only produces eigenvectors and eigenvalues associated with the Γ irrep or subirrep.

In implementing the filter diagonalization of *Ĥ*_bend_^(2B)^ one requires the evaluation of the repeated effects of operating with *Ĥ*_bend_^(2B)^ on |Ψ_Γ_⟩. We perform all these required evaluations by computing matrix-vector products, with the matrix elements of the components of *Ĥ*_bend_^(2B)^ computed beforehand.

#### 2.7.1 Computing the Matrix Elements of *T̂*_bend_

The matrix elements of *T̂*_bend_ are of two types, one-body and two-body. The one-body part involves the operator
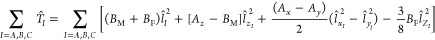
34The two-body part of *T̂*_bend_ is given by
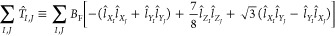
35where (*I*, *J*) = (*A*, *B*), (*B*, *C*), (*C*, *A*). We evaluate the matrix elements of these operators by first computing, analytically all the relevant one-body matrix elements of the operators , , , , , , and  in the set of hindered-rotor states. For any given one of these operators, , the relevant matrix elements are given by
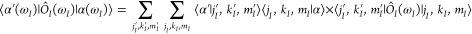
36and all of the ⟨*j*_I_^′^, *k*_I_^′^, *m*_I_^′^|*Ô*_*I*_(ω_*I*_)|*j*_*I*_, *k*_*I*_, *m*_*I*_⟩ are available analytically.

For the matrix elements of the two-body part of *T̂*_bend_ (eq 35) we use the fact that the ⟨α′(ω_*I*_), β′(ω_*J*_)|*T̂*_*I*,*J*_(ω_*I*_, ω_*J*_)|α(ω_*I*_),β(ω_*J*_)⟩ can easily be written as a sum over products of already-evaluated (eq 36) single-body matrix elements. Considerations of symmetry also significantly reduce the computational effort associated with obtaining all the required ⟨α′β′|*T̂*_*I*,*J*_|αβ⟩. In particular, it can be easily shown (see the Section S4) that these matrix elements are only nonzero if (a) *p*_α_*p*_β_*p*_α′_*p*_β′_ = +1, (b) *q*_α_ = *q*_α′_, and (c) *q*_β_ = *q*_β′_.

#### 2.7.2 Computing the Matrix Elements of the *V*_bend_^(2B)^(ω_*I*_,ω_*J*_) and *V*_bend_^(2B[3B])^(ω_*I*_,ω_*J*_)

The procedures for computing the matrix elements of *V*_bend_^(2B)^(ω_*I*_,ω_*J*_) and *V*_bend_^(2B[3B])^(ω_*I*_,ω_*J*_) are the same. As such, we provide the details below only for the *V*_bend_^(2B)^(ω_*I*_,ω_*J*_) matrix-element calculations. Replacing *V*_bend_^(2B)^(ω_*I*_,ω_*J*_) with *V*_bend_^(2B[3B])^ in what follows yields the process by which the *V*_bend_^(2B[3B])^(ω_*I*_,ω_*J*_) matrix elements are obtained.

Just as with the *T̂*_*I*,*J*_ matrix elements, the ⟨α′,β′,γ′|*V*_bend_^(2B)^(ω_*I*_,ω_*J*_)|α,β,γ⟩ matrix elements are diagonal with respect to the indices associated with ω_*K*_, *K* ≠ *I*, *J*. In addition, one has by symmetry
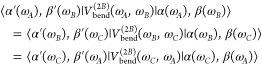
37and ⟨α′, β′|*V*_bend_^(2B)^|α, β⟩ = 0, if *p*_α_*p*_β_*p*_α′_*p*_β′_ = −1, or *q*_α_ ≠ *q*_α′_, or *q*_β_ ≠ *q*_β′_.

To evaluate the nonzero matrix elements, rather than working with a quadrature-grid representation of the functions involved, we choose instead to work with an expansion of *V*_bend_^(2B)^(ω_*I*_,ω_*J*_) over symmetric-top eigenfunctions (normalized Wigner matrix elements):
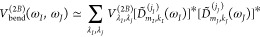
38where λ_*I*_ ≡ (*j*_*I*_, *k*_*I*_, *m*_*I*_). The extent of the expansion is defined by *j*_max_^expand^: All the Wigner-matrix-element products corresponding to *j*_*I*_, *j*_*J*_ ≤ *j*_max_^expand^ are included (initially). The expansion coefficients are given by
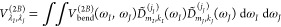
39which we compute by quadrature.

The matrix elements of interest are then given by
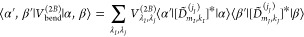
40The one-body matrix elements on the rhs of eq 40 can be computed analytically by making use of eq 21 above and eqs 3.118 of ref (39). Thus, for example, one has
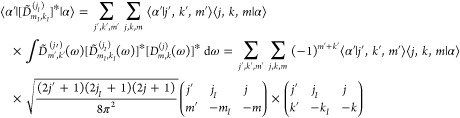
41where the quantities in large parentheses are 3-*j* symbols. To simplify notation going forward, we define
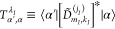
42so that eq 40 can be re-expressed as
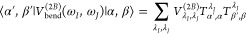
43

One can further exploit symmetry to reduce the number of terms that need to be computed in eq 43—see Section S5. Specifically, only those terms in the summation on the rhs of that equation that correspond to even *k*_*I*_ and *k*_*J*_ values are nonzero. Further,
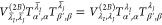
44where  and . eq 44 can be used to reduce the number of terms in eq 43 by about a factor of 2.

#### 2.7.3 Matrix-Vector Products

In computing the matrix-vector products required to implement CFD
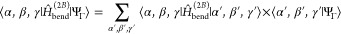
45we make use of the structure of  (see eq 30) to significantly reduce the computational cost. In particular, we multiply separately the  vector with the matrices corresponding to , *I* = *A*, *B*, *C*, and , (*I*, *J*) = (*A*, *B*), (*B*, *C*), (*C*, *A*), respectively, and then add the results (we will henceforth abbreviate [*V*_bend_^(2B)^(ω_*I*_, ω_*J*_)+ *V*_bend_^(2B[3B])^(ω_*I*_, ω_*J*_)] as *V*_*I*,*J*_). Thus, instead of having to loop over six indices, as suggested by eq 45, the multiplications involving the one-body operators can be effected by looping over only four indices, and those involving the two-body operators by looping over only five. In addition, the relevant operator matrices in our symmetry-specific CFD runs are substantially reduced relative to the sizes they would have if symmetry were not taken into account. For example, for the  irrep, the only part of the full  matrix that is relevant is that for which the basis states have H-exchange eigenvalues . (See Table 2.) Further, that much-reduced matrix, can be block-diagonalized into two blocks–those for which  – which further reduces the cost of the multiplication by about a factor of 2. Finally, there are restrictions on the values of the two indices (in the case of the one-body operators) or the single index (in the case of the two-body operators) that do not correspond to any of the operator-matrix indices but must be looped over to effect the matrix-vector multiplication. An example is the index γ in the multiplication
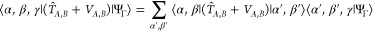
46eq 46 must be evaluated for numerous values of γ. However, these values are limited by symmetry. For the  case referred to above, for example, the only relevant values of γ in eq 46 are those for which  (only such  contribute to the  basis). In addition only  corresponding to  are relevant for the blocks of the  matrix corresponding to .

Finally, for all of the *A* irreps one can further reduce the computational cost of matrix-vector operations by virtue of the following relations, which we prove in Section S6:
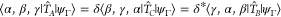
47and
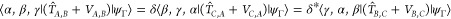
48where  for Γ equal to *A*_1_-type, *A*_2_-type, and *A*_3_-type irreps, respectively. As a result of these relations, one need only compute say,  and  for all relevant  to obtain trivially the effects of operating with all of the other components of  on , as well.

### 2.8 Diagonalization of the Full *Ĥ*_bend_

With the eigenvectors and eigenvalues of *Ĥ*_bend_^(2B)^ computed, we then diagonalize *Ĥ*_bend_ in a truncated basis of low-energy *Ĥ*_bend_^(2B)^ eigenvectors. Denoting the latter associated with a given *G*_48_ irrep or subirrep Γ as 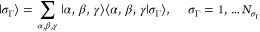
49one has for the matrix elements of *Ĥ*_bend_ for the Γ block of the Hamiltonian (which is block-diagonal with respect to the Γ):
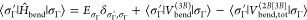
50where  is the eigenvalue of   with respect to . Clearly, the main tasks in diagonalizing  in this scheme are the evaluation of the  and  matrix elements in the  bases.

#### 2.8.1 Evaluation of the *V*_bend_^(3B)^ Matrix Elements

The size of the 9D grid required to evaluate by quadrature the integrals associated with the matrix elements of *V*_bend_^(3B)^ in eq 50 is of order 2 × 10^10^ points. (For example, with *j*_max_ = 12 for the primitive, single-site basis set (see eq 20), the ω_*I*_ angle grid for that site should ostensibly consist of 14 × (26)^2^ = 9464 points. Thus, the full 9D angle grid for this *j*_max_ should consist of (9464)^3^ points. Symmetry can be used to reduce this size by about a factor of 48.) Such evaluation requires (a) computing and storing  on such a grid, (b) transforming all the relevant  (on the order of 5 × 10^2^ in number) to the grid and storing the results, and (c) performing sums over the grid points for all of the required  matrix elements. The storage requirements to implement this approach are very demanding. Given this, we have chosen to work with  represented as an expansion over products of normalized Wigner matrix elements in order to evaluate the necessary matrix elements.

Analogous to eq 38 for the two-body PES terms, one can write for 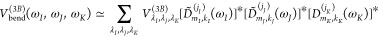
51where
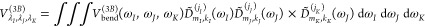
52We evaluate these coefficients by quadrature. The matrix elements in eq 50, making use of the definition in eq 42, can then be written as
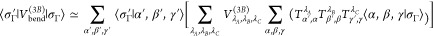
53

The significant advantage of this approach is that the memory needed to store the terms involved in eq 53 is readily available with current technology. However, the evaluation of eq 53, which has to be done on the order of 10^5^ times, is daunting, as it requires summing over nine indices each of which runs over ca. 100 values or more. Thus, one seeks ways to evaluate the summations efficiently and, if possible, reduce the number of terms in those summations.

As to the former, we first evaluate ∑_α,β,γ_*T*_α′,α_^λ_A_^*T*_β′,β_^λ_B_^*T*_γ′,γ_^λ_C_^⟨α, β, γ|σ_Γ_⟩ for all necessary α′, β′, γ′, an initial set of λ_*A*_, λ_*B*_, λ_*C*_ values, and an initial |σ_Γ_⟩. This can be done efficiently in a now-standard type of procedure, as follows. First,
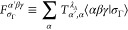
54is evaluated for all α′, β, γ. Then
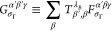
55is evaluated for all α′, β′, γ. Finally,
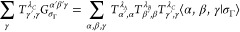
56is evaluated for all α′, β′, γ′. The cost of this procedure goes as 3*n*^4^ (rather than *n*^6^) where *n* is the number of single-site hindered-rotor eigenstates used to construct the symmetry-specific 9D basis. We multiply each of the quantities given by eq 56 by , store the result, and repeat the entire process for the next set of  values. These new values of  (for all α′, β′, γ′) are added to the first ones, and the whole procedure is repeated until all of the  values have been covered. The end result is the evaluation of
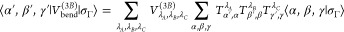
57for all relevant . These quantities are then contracted with the  for all  to obtain  for all  The entire process is repeated for each , and all required matrix elements are thus obtained. This algorithm is easily parallelized, and we have performed the  matrix-element calculations required for this work by using open-MPI running on 60 processors.

In respect to minimizing the number of terms in the  summation on the rhs of eq 57 we do two things. First, we exploit symmetry. As detailed in Section 2.10 below, the expansion we use for  (see eq 51) leads to  summations that nominally consist of  terms. However, as we show in Section S7, when Γ is an *A* irrep this number can be reduced in eq 57 by about a factor of 48 (without approximation), and when Γ is a *T* subirrep, it can be reduced by about a factor of 16. Second, we use the fact that many of the  expansion coefficients (after aggregating by symmetry) are very small relative to the ones that contribute the most to the  expansion. In particular, we include only those  values in eq 57 that correspond to expansion coefficients with magnitudes above a predetermined threshold. This approximation reduces the number of  terms in eq 57 to the neighborhood of 10^4^ to 10^5^. We address the effect of this approximation on the accuracy of the 9D eigenvector results below in Section 2.10.

#### 2.8.2 Evaluation of the *V*_bend,tot_^(2B[3B])^ Matrix Elements in the |σ_Γ_⟩ Bases

Compared to the evaluation of the  matrix elements, computing the  is much less expensive. The relevant matrix-element pieces in the primitive hindered-rotor basis, , are already available from the procedure used to diagonalize . One can immediately use these to obtain the desired quantities. For example,
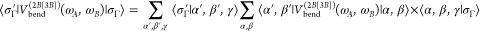
58Similar relations are easily obtained for the matrix elements of  and for those of .

#### 2.8.3 Eigenstates of *Ĥ*_bend_

With the matrix elements of *Ĥ*_bend_ in a given irrep/subirrep |σ_Γ_⟩ block computed, we then diagonalize that matrix directly. We label the resulting eigenvectors and corresponding eigenvalues |κ_Γ_⟩ and *E*_κ_Γ__ (κ_Γ_ = 1, ..., *N*_κ_Γ__), respectively. The eigenvectors can be expressed as expansions over the |σ_Γ_⟩ basis states or over the hindered-rotor |α, β, γ⟩ basis:
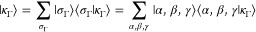
59where
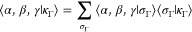
60

### 2.9 Calculation of Electric-Dipole Transition Moments

With the eigenvectors of *Ĥ*_bend_ in hand there are numerous ways by which one can characterize the corresponding states. Space limitations preclude us from doing this in anything like comprehensive fashion in this work. However, one thing we do include here is a calculation of dipole-moment matrix elements corresponding to transitions from lower-energy bend eigenstates to higher-energy ones. To perform these calculations we assume that the trimer dipole operator is the vector sum of the permanent dipoles of the three constituent monomers. In that case, the trimer’s dipole operator is given by , where μ is the magnitude of water monomer’s ground-state permanent dipole moment.

We are interested in the components of  along the “global” trimer-fixed axes  (defined in ref (38), Section 3). We thus need the direction cosines between the global axes and the three sets of local axes , , with respect to which the bend coordinates, ω_*I*_, are defined. The direction cosines, which we denote as , where ,  correspond to , , and , respectively, and  correspond to , , and , respectively, are easily determined from the axis definitions. One then has for the component of  along the *k*th global axis
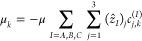
61where (*ẑ*_*I*_)_1_ = sin θ_*I*_ cos ϕ_*I*_, (*ẑ*_*I*_)_2_ = sin θ_*I*_ sin ϕ_*I*_, and (*ẑ*_*I*_)_3_ = cos θ_*I*_. The matrix element of μ_*k*_ between initial bend state  and final bend state  can then be expressed as
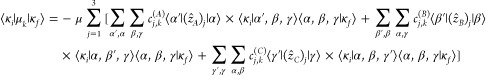
62eq 62 can be readily evaluated by first computing by quadrature the single-monomer, hindered-rotor matrix elements  for all , and then using the results in eq 62. We present the results of these calculations in the form of the simulated absorption spectrum of the trimer shown in Section 3.2.3, wherein the intensity of each  transition is taken as .

### 2.10 Summary of Computational Parameters

We list here the values of the various parameters used to produce the results presented below. We took the H and O masses to be 1837.153 au and 29 156.946 au, respectively. The OH monomer bond distance was set at 1.84371 bohrs, and the monomer bond angle at 104.43^◦^, corresponding to the averaged structure of the isolated water monomer in its ground state.^41^ The intermonomer cm-to-cm distance was taken to be  bohrs, which corresponds to its expectation value in ground state of the 3D frame Hamiltonian of the water trimer. The value chosen for *j*_max_ in computing the hindered-rotor states was 12. For *N*_HR_ we used 200. (We also did limited calculations with *N*_HR_ = 240 and 280 in order to check convergence.) The number of hindered-rotor functions of each of the four possible  symmetries equaled . The  bases employed in the diagonalization of  consequently consisted of 500,000 functions per irrep/subirrep. In diagonalizing  the  basis sets all consisted of the 500 lowest-energy functions of a given irrep/subirrep.

Finally, the parameters relevant to the expansion representations of , , and  are given in Table 3. In the Table, *j*_max_^expand^, the largest *j*_*I*_ value in the expansion, defines the total possible number of nonzero terms, *N*_max_^expand^, in each expansion. The actual number of expansion terms relevant to any given calculation, *N*^expand^, depends on the choice of the coefficient-threshold value, , the value below which expansion terms (aggregated by symmetry) were set to zero. Last, for each set of expansion parameters we quote the root-mean-squared deviation, Δ*V*, of the expansion-computed potential-energy values from those computed from the relevant PES function.

**Table 3:** Details of the Expansion Representations of , , and

			/cm^–1^	*N*^expand^	Δ*V*/cm^–1^
	10	793,881	0.01	511,315	1.05
	10	793,881	0.01	196,271	0.02
	6	12,326,391	10.0	427,433	2.16

## 3 Results and Discussion

### 3.1 Results of the Diagonalization of *Ĥ*_bend_^(2B)^

The eigenvectors of *Ĥ*_bend_^(2B)^, the , are useful primarily as constituents of an efficient basis with which to diagonalize the full . For that purpose we employ up to 500  for each *G*_48_ irrep, Γ. For the *A* irreps this includes states with energies up to about 1300 cm^–1^ above the  ground state. For the *T* irreps the states extend up to about 1000 cm^–1^ above that ground state. There seems little purpose to enumerate all of these states here. However, there is some value in examining some of the characteristics of those states with the lowest energies, as these states contribute overwhelmingly to the low-energy  eigenstates that interest us. As such, we present a listing of the 30/80 lowest-energy  eigenvalues of each of the *A/T G*_48_ irreps in Tables S1–S8. In those Tables, in addition to energies, we also present information pertaining to the efficiency of the hindered-rotor basis in covering the space of these eigenstates. Specifically, we sum up the total contribution of the 100 highest-contributing symmetry-adapted HR basis functions to each eigenstate. We find that these contributions are routinely >80%. Bearing in mind that the basis-set sizes that we use for the irrep/subirrep are of order 10^5^ states, one sees that a small fraction of each basis contributes overwhelmingly to each low-energy eigenstate.

It is also valuable to consider some of the lowest-energy  eigenstates by grouping them together according to the *G*_6_ torsional levels to which they correspond. Figure 4 shows the torsional level structure and the bifurcation substructure, together with tunneling splitting parameters corresponding to . For torsional levels of *A*_1_^±^*G*_6_ symmetry the eight pertinent member states are of , ,  (triply degenerate) and  (triply degenerate) *G*_48_ symmetry. Hence, there are four distinct energies corresponding to such levels. For torsional levels of  symmetry the 16 member states are of  (doubly degenerate),  (doubly degenerate),  (triply degenerate),  (triply degenerate),  (triply degenerate), and  (triply degenerate) symmetry. Hence, for these torsional levels there are six distinct energies. The substructure of these torsional levels arises from the effect of non-negligible bifurcation tunneling in the species, and the splitting patterns due to such tunneling have been extensively investigated elsewhere.^13,14,26,42^ Given that  is invariant to the operations of *G*_48_, one would hope that our -eigenstate results would conform to these patterns. The principal unknown in this regard is whether the separate  diagonalizations corresponding to different irreps converge in similar fashion, such that small computed energy differences between the states of different irreps belonging to the same torsional level can be trusted to be meaningful.

Tables 4 and 5 present results from our calculations that speak to this issue. Table 4 lists the energies of the tunneling components for the lowest-four levels of *A*_1_^±^ and of *A*_1_^−^*G*_6_ symmetry. The splitting of such levels should conform to a pattern defined by a single energy parameter, β, wherein the energies of the , *T*_*u*_, *T*_*g*_, and  component states are given, respectively, by , , , , relative to the average energy of the level (see, e.g., Table 5 of ref (42), and Table 4 of ref (11). In Table 4 we present the torsional energies and the value of β fitted to the calculated energies for each level and the root-mean-squared deviation (“rms”) of those energies from the ideal pattern implied by the value of β. One notes that for these levels the computed splitting patterns conform very well to those expected from theory.

**Table 4:** Computed Bifurcation-Tunneling Splittings of Low-Energy  Torsional Levels of

	a	b				β	rms
	0.007	–200.0	–66.7	66.7	200.1	133.4	0.01
	145.48	77.9	25.9	–26.0	–77.8	–51.9	0.02
	207.68	–135.2	–45.0	45.1	135.1	90.1	0.07
	271.60	–2599.4	–866.6	866.2	2599.8	1733.0	0.20
	89.86	102.3	34.2	–34.0	–102.4	–68.2	0.08
	151.37	774.1	257.9	–258.1	–773.8	–516.0	0.13
	154.52	–531.1	–177.6	176.8	532.0	354.4	0.43
	243.81	852.2	279.9	–286.1	–846.0	–566.1	3.08

a Average energy of the torsional level in cm^–1^ relative to the  ground state at −4012.162 cm^–1^.

b All splitting energies are relative to the average energy of the torsional level. The splittings, as well as the β and rms values, are in MHz. The definitions of β and rms are given in the text.

**Table 5:** Computed Bifurcation-Tunneling Splittings of Low-Energy  Torsional Levels of

	a	b						β	δ	rms
	68.34	–7.1	7.1	–89.8	94.5	–94.5	89.7	4.7	92.1	0.01
	150.54	–1295.3	1295.9	–414.5	1277.6	–1277.4	413.1	863.7	845.6	0.41
	229.60	–247.7	247.7	–553.6	718.7	–717.9	552.7	165.2	635.7	0.32
	276.15	–2333.1	2333.2	–588.5	2144.2	–2144.7	588.7	1555.5	1366.5	0.19
	24.01	49.0	–49.0	–76.1	43.4	–43.5	76.1	–32.7	59.8	0.02
	193.69	–75.6	75.3	–587.2	637.7	–636.9	586.8	50.3	612.1	0.26
	215.29	–420.8	420.8	–276.6	557.1	–556.2	275.7	280.5	416.4	0.33
	271.04	2527.3	–2540.0	–3254.4	1578.0	–1549.4	3251.2	–1689.1	2408.2	8.48

a Average energy of the torsional level in cm^–1^ relative to the  ground state at −4012.162 cm^–1^.

b All splitting energies are in MHz and are relative to the average energy of the torsional level.

Table 5 lists the energies of the tunneling components for the lowest-four levels of *A*_2/3_^+^ and *A*_2/3_^−^*G*_6_ symmetry. The splitting of these levels is expected from theory to depend on two energy parameters, β and δ, and to conform to the pattern , , , , ,  for the , *T*_*u*_, *T*_*u*_, *T*_*g*_, *T*_*g*_, and  component states, respectively.^42^ We have derived from the data values of β and δ for each level and have compared the ideal level structure based on these values with the computed splittings. The β and δ values, as well as the rms deviation of the computed energies from the ideal splitting pattern, are given in Table 5 for each level. Here, too, one notes the good agreement between the computed splittings and the pattern expected from theory.

For computed  levels higher in energy than the ones pertaining to Tables 4 and 5 there is a trend toward less conformity with the theoretical splitting patterns referred to above. There are two likely reasons for this. First, the theoretical patterns rest on several assumptions that might be reasonably expected to break down with increasing excitation energy.^42^ Indeed, experimental results^11^ indicate significant deviations from those patterns for bend levels at excitation energies larger than those listed in Tables 4 and 5. Second, it is also possible that the similar rates of convergence of the  calculations which hold for the eigenenergies of the lowest-energy torsional levels, do not apply for the states of higher-energy levels.

While our principal aim here is to obtain and, ultimately, assess the results of the diagonalization of the full , it is perhaps of some interest to compare select  results with available experimental ones. The latter includes the excitation energies corresponding to the ,  and  torsional energy levels, and the bifurcation-tunneling-splitting parameters corresponding to the *A*_1_^+^(1) → *A*_1_^–^(1), *A*_1_^+^(1) → *A*_2/3_^+^(1), and *A*_2/3_^–^(1) → *A*_2/3_^+^(1) far-infrared bands.^43^ In respect to the former, one sees from Table 7 that the computed excitation energies are too large by about 3% to 6% (1.3 to 2.8 cm^–1^). We shall see below that the corresponding discrepancies are reduced significantly when the full  eigenvalues are considered. As for bifurcation-tunneling-splitting parameters—the quartet splittings (i.e., ) and  values derived from  transitions—one sees from Table 8 that the computed values agree in respect to order-of-magnitude with values obtained from experiment.

Finally, it is pertinent to consider in more detail the degree of convergence of the  results. We have assessed this by computing the  level structure for three different primitive-basis-set sizes: the 500,000-function basis set corresponding to the results quoted above, along with basis sets containing 864,000 and 1,372,000 functions. Table S9 presents the relevant results. In brief summary, the absolute energies of the computed ground state decrease by about 0.09 cm^–1^ in going from the smallest basis to the largest, whereas such decrease increases to about 0.74 cm^–1^ for state #20 (Δ*E* ≃ 517 cm^–1^), and to about 1.3 cm^–1^ for state #40 (Δ*E* ≃ 653 cm^–1^). Essentially all of the  results presented below were obtained by using  eigenstates corresponding to the 500,000-function bases. Hence, we estimate convergence errors of about 1 cm^–1^ (at least in respect to limitations imposed by the size of the primitive basis) for eigenenergies of  having Δ*E* ≃ 600 cm^–1^, with such error being an order of magnitude smaller for states nearer the bottom of the level structure.

### 3.2 Results of the Diagonalization of *Ĥ*_bend_

#### 3.2.1 Convergence Attributes

Extensive listings of the computed eigenenergies of *Ĥ*_bend_ are compiled in Tables S10–S17. The results correspond to the 30 lowest-energy eigenstates for each of the eight *A* irreps (up to about Δ*E* = 600 cm^–1^) and the 80 lowest-energy eigenstates for each of the four *T* irreps (up to about Δ*E* = 590 cm^–1^). In addition to the Δ*E* values, there is also listed for each state in the Tables the basis-state norm,  corresponding to the largest basis-state (|σ_Γ_^max^⟩) contributor to each eigenstate (|κ_Γ_⟩). The latter numbers, particularly for the lowest-energy states, are routinely greater than 0.9, testament to the efficiency with which the -eigenstate bases cover the space of the low-energy  eigenstates.

One universal feature of the  eigenvalues is that each is roughly 50–60 cm^–1^ more positive than the  eigenvalue of the basis state that dominates in contributing to it. This is due in part to the fact that the diagonal matrix elements, , that contribute to the matrix of  in the  basis are much larger in amplitude (typically by a factor close to 100) than the off-diagonal elements of the matrix. Hence, the diagonal elements dominate in determining the change from  eigenvalues to  eigenvalues. In addition, the  values are all positive and mostly in the range of 50 to 60 cm^–1^. That they are positive is attributable to the fact that  generally overestimates the attractiveness of (is more negative than) the true 3-body term at a given 9D point because the former potential function is defined with one monomer fixed in a low-energy position (see eq 32). The 50–60 cm^–1^ diagonal elements constitute a measure of the extent of that overestimation.

It is worth pointing out that we have tested an algorithm similar to that employed here to diagonalize , but with eigenstates of  without the  term included used as the basis. The efficiency of that algorithm is notably worse than the present one, and significantly larger  bases are required to achieve a given level of convergence. The upshot is that the complication of including  in the algorithm is more than compensated for by the enhanced performance that results from its inclusion.

**Figure 3:**
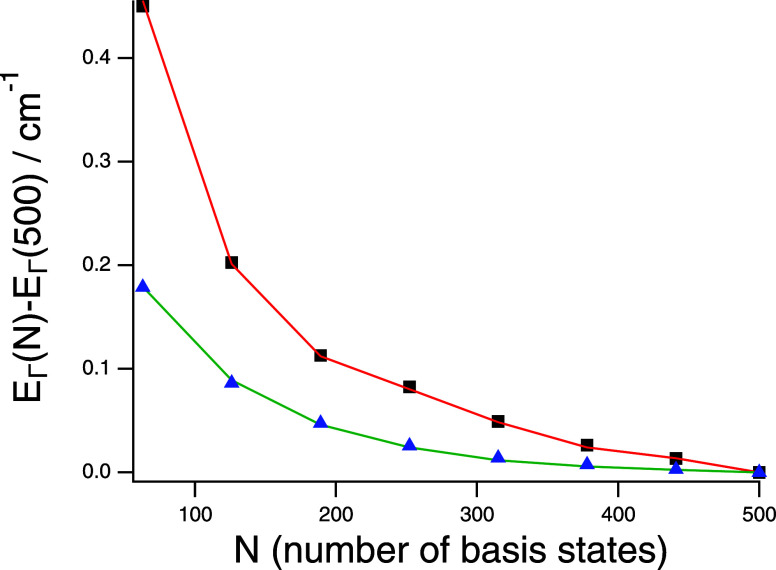
Computed  energy eigenvalues of the states of the *A*_1_^+^(1) *G*_6_ level of  vs the size, *N*, of the basis. The energies corresponding to a given state are all relative to the computed energy of that state for *N* = 500:  state (green line),  state (blue diamonds),  state (red line),  state (black squares).

Figure 3 presents information pertaining to the convergence behavior of  eigenstates with respect to the sizes of the irrep-specific  basis sets. The figure pertains to the states comprising the ground-state torsional level: , , , and  and shows plots of the difference of the computed energy of each state for a given basis set size, *N*, minus the corresponding energy for *N* = 500 vs *N* (i.e.,  vs *N*). There are two points to note about these data. First, the *A* states are quite well-converged at *N* = 500, but the *T* states are slightly less so. Second, the *A* states have very close to the same convergence rates, as do the *T* states, but those rates are different for the different classes of irreps. Similar behavior is exhibited by the *A* and *T* states comprising the other low-energy torsional levels. Such behavior can be understood by noting that the  level structures for  and  are very similar, as are those for  and , but the *A* and *T* level structures are significantly different from one another (see Tables S1–S8). Given this, one clearly cannot obtain meaningful bifurcation-splitting patterns for the ground-state and other low-energy levels by blindly using differences in the computed  energies as they were used above with the  energies. However, one can have some confidence in the computed energy differences between the *A* states of a given level and those between the *T* states of a given level (at least to within an order of magnitude). And, one can use those energy differences to obtain significant features of the splitting patterns, as we do in what follows.

#### 3.2.2 Energetics of the *Ĥ*_bend_ Level Structures

**Figure 4:**
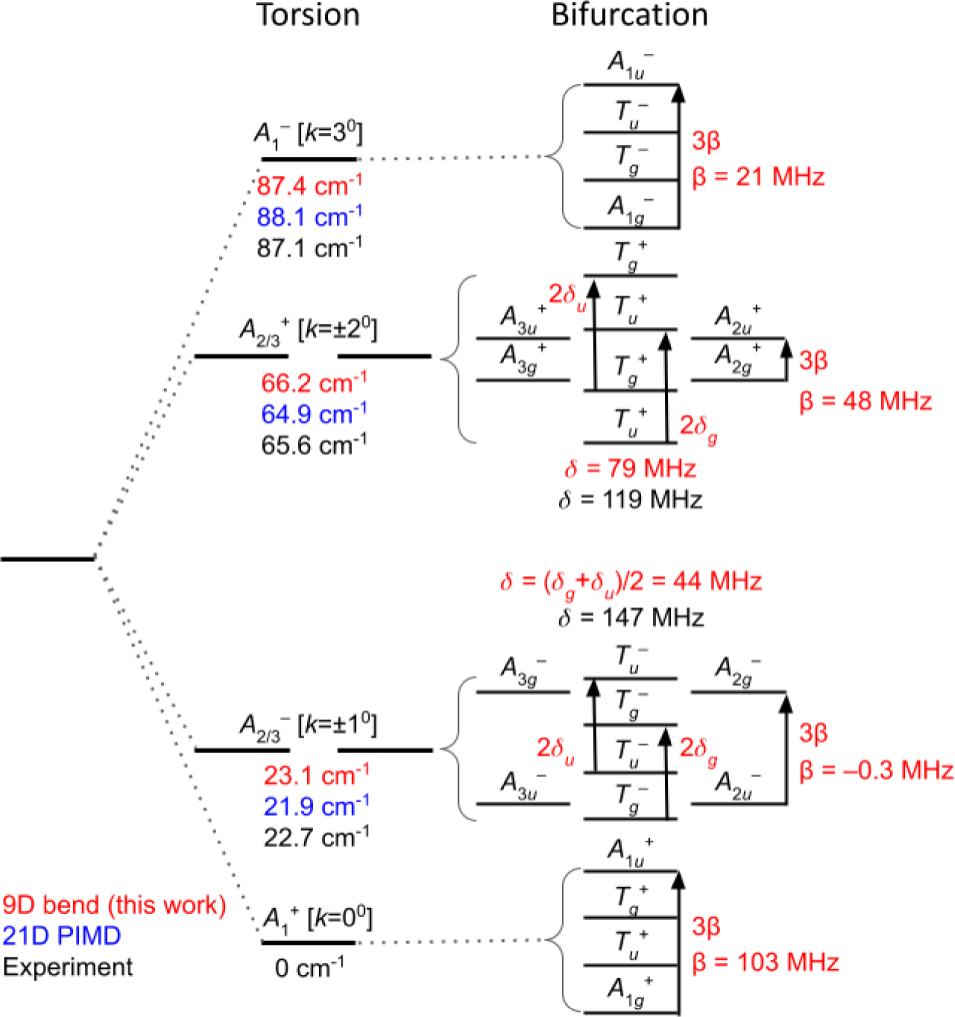
Calculated (9D  in this work and 21D PIMD^29^) and experimental^43^ torsional and bifurcation tunneling splittings in the ground vibrational state of the water trimer.

Table 6 presents the  results in a manner similar to the presentation of the low-energy  results of Tables 4 and 5, while Figure 4 shows the torsional states and bifurcation splitting parameters of the ground vibrational state. The Tables list the  of the *G*_6_ levels and the values of bifurcation-splitting parameters associated with each level (β in the case of the  levels and β and δ in the case of the  levels). To obtain the  value for a given  level we computed , where  and  are the energies of the  and  states within that level and  cm^–1^ is the ground-state energy. The bifurcation-splitting parameter β for such levels was obtained by using . Similarly, for the  levels we used  and . For these latter levels δ was also computed from the average splittings of the  and the  states within the level: , where  and , and , ,  and  are the two  and two  energies, respectively, that contribute to the level.

**Table 6:** Energy Characteristics of the Lowest-Energy Torsion/Libration Levels of

*G*_6_ irrep	*N*	/cm^–1^^a^	β/MHz	δ/MHz	b/MHz
	1	0.005	102.9	–	–
	2	146.61	–32.5	–	–
	3	207.03	58.0	–	–
	4	274.31	1046.1	–	–
	5	277.51	358.4	–	–
	6	316.19	3027.0	–	–
	1	23.14	–0.3	44.3	2.5
	2	193.40	55.8	440.5	13.0
	3	216.30	233.7	367.4	17.7
	4	273.86	–1236.0	1784.4	19.4
	5	310.83	–3741.7	4240.2	21.9
	6	324.59	741.2	748.6	0.9
	1	66.16	47.7	79.2	11.9
	2	151.52	669.0	600.9	4.5
	3	229.54	148.2	535.8	4.7
	4	280.19	1075.2	935.9	9.8
	5	314.26	633.2	661.7	55.4
	6	321.18	–201.4	1188.3	11.1
	1	87.37	20.7	–	–
	2	152.08	–404.1	–	–
	3	154.35	292.1	–	–
	4	244.88	–428.4	–	–
	5	318.02	872.7	–	–
	6	328.06	–1650.9	–	–

a Energies relative to the ground-state energy of −3968.752 cm^–1^.

b .

The assignment of the levels in Table 6 is not trivial for any but the lowest-energy one of each symmetry. These latter clearly correspond to the four pseudorotational torsional levels created by the hydrogen-flip tunneling splitting of the ground vibrational state–i.e., , , , and 3^0^. However, the floppiness of the trimer, with its 48 equivalent and accessible minimum-energy geometries, presents significant challenges to the assignment of the higher-energy states, even with the corresponding (highly delocalized) eigenstates in hand. In fact, the task of assignment is involved enough that we choose to take it up in a later paper. That said, there are several further points about the  results that can be considered here.

First, one can compare computed energy levels with experimental and other computational results. The energies of the three lowest-energy excited pseudorotational levels are well-characterized by experiment. Moreover, those energies have been computed in full dimensionality by Zhang et al.,^29^ by using path-integral molecular dynamics with the PES that they calculated, and which is employed in this work as well. Table 7 and Figure 4 present such comparisons. One notes that the  results already agree with the measured data to within 3–6% (1.3–2.8 cm^–1^), while the discrepancy decreases to 0.3–2% (0.3–0.6 cm^–1^) for the  results. Note that this excellent agreement is partially fortuitous and probably involves error cancellations, since our 9D model gives slightly better agreement with the measured data than the 21D PIMD simulation by Zhang et al.^29^ We are currently investigating this possibility by extending calculations on the trimer from the 9D bend problem treated here to the full 12D intermolecular (rigid-monomer approximation) problem.

**Table 7:** Comparison Between Calculated and Experimental /cm^–1^ Results

*G*_6_ level	9D a	9D a	21D PIMD^b^	Expt.^c^
[]	24.0	23.1	21.9	22.7
[]	68.3	66.2	64.9	65.6
[]	89.9	87.4	88.1	87.1

a This work.

b Reference^29^

c Reference (43)

Second, one can compare computed bifurcation-splitting parameters with those obtained from experiment. We do this in Table 8 and Figure 4. As with the  results one sees that in this regard the full  results are, at best, in semiquantitative agreement with those from experiment. That said, the variation from level to level in the computed bifurcation splittings (see Table 6) is considerably larger than the differences between the computed and experimental results in Table 8. In short, there is some promise in the idea that one can make use of the present computational results to correlate the magnitude of the bifurcation splittings associated with a given level with the nature of the vibration to which that level corresponds. We pursue this idea in a follow-up work focused on the assignment of the bend states computed here.

**Table 8:** Comparison Between Calculated and Experimental Bifurcation-Splitting Parameters, Given in MHz

	9D a	9D a	Expt.^b^
Quartet splittings			
	202	82	289.0
	129	24	253.46
	37	35	38.88
δ values			
	60	44	147
	92	79	119

a This work.

b Reference (43)

#### The Absorption Spectrum Calculated from the Eigenstates of 

3.2.3

Calculation of the trimer’s bend eigenstates allows us to make direct spectroscopic comparisons with experiment. In particular, we compute the absorption spectrum of the water trimer by using the results of Section 2.9, with the goal of comparing it to the FIR spectrum of the trimer in helium nanodroplets, measurements made at the ultracold temperature of 0.37 K.^25^ At that temperature all transitions in the measured spectrum originate from the  torsional level. Electric-dipole selection rules allow the  and  parallel transitions, while the perpendicular transitions are ,  and  In terms of *G*_6_ symmetry, the selection rules are  for the parallel transitions and  for the perpendicular transitions.

**Figure 5:**
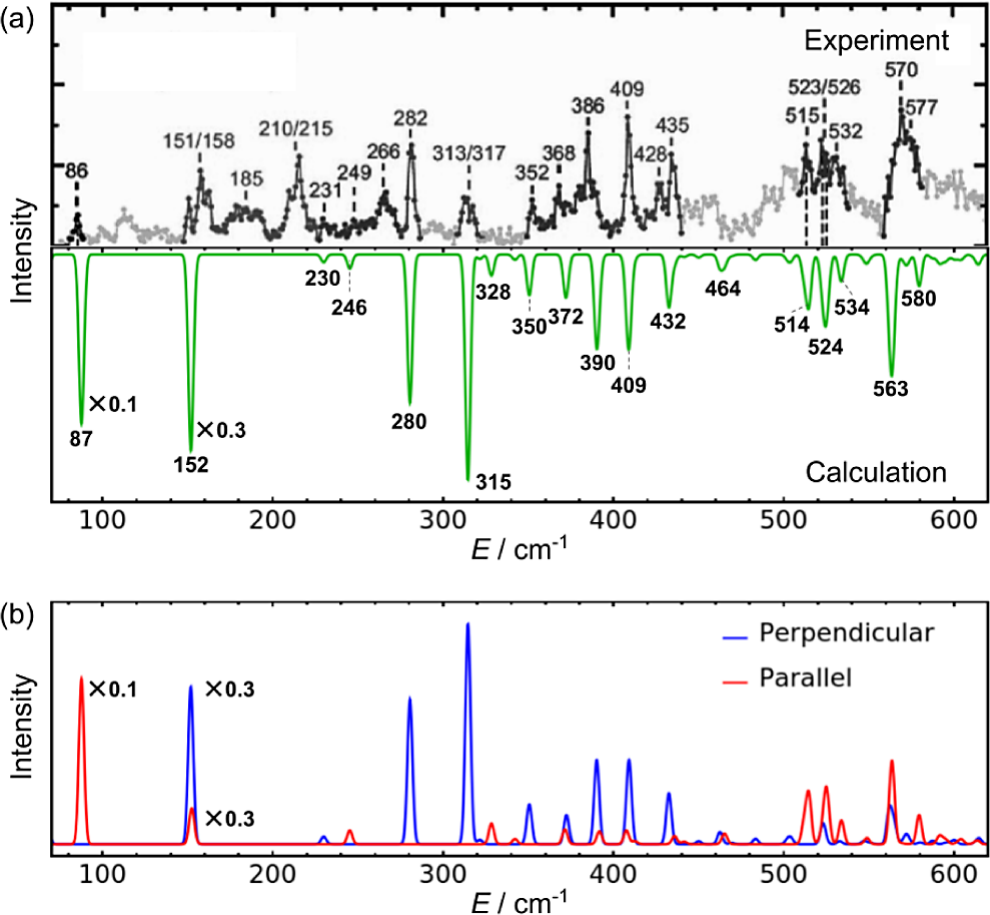
(a) Top: measured FIR spectrum of the water trimer by the Havenith group. Adapted with permission from ref. (25) Copyright 2024 John Wiley and Sons. (a) Bottom: spectrum calculated from the 9D eigenstates of  in this work. Note that the calculated spectrum includes only the intermolecular bending transitions, while the measured spectrum contains intermolecular stretching transitions as well. (b) Parallel and perpendicular components of the calculated spectrum.

**Figure 6:**
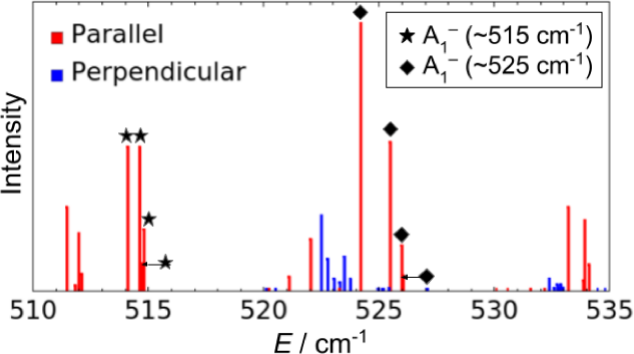
Calculated bend transitions in the range of the spectrum measured by the Saykally group.^31^ The transition quartets marked by stars and diamonds correspond to bifurcation components within two distinct excited  torsional levels. See text for details.

Calculated spectra are shown in Figures 5 and 6. Each is obtained by using
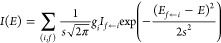
63where  is the transition energy of the  transition,  is the transition intensity (see Section 2.9), *s* = 1.5 cm^–1^ for Figure 5 and *s*→ 0 for Figure 6 is a line width parameter, and *g*_*i*_ is the nuclear spin statistical weight of . The nuclear spin statistical weights are the following: , ,  and 

In Figure 5a, the calculated spectrum is compared to the experimental one from ref (25), and in Figure 5b the parallel/perpendicular character of the bands is shown. It is apparent at a glance that the match between the two spectra in Figure 5a is impressive both visually and in terms of alignment of the individual computed and measured peaks over the entire range of the measured FIR spectrum. Such a level of agreement is remarkable given the fact that the present calculations do not include the intermolecular stretching vibrations of the trimer. From this, one can conclude that (1) most of the transitions visible in the experimental FIR spectrum arise from the excitation of what are primarily the bending vibrations of the trimer, (2) intermolecular bending and stretching vibrations are weakly coupled, (3) the PES by Zhang et al.^29^ is of high accuracy, and (4) the rigid-monomer approximation is apparently adequate to the purpose of characterizing the intermolecular level structure of the trimer.

Notwithstanding the above, it is evident from Figure 5a that several peaks present in the measured FIR spectrum, e.g., at 185 cm^–1^, 210/215 cm^–1^, and at 266 cm^–1^, are missing from the computed spectrum. Given the frequencies involved, it is highly likely that these transitions are associated with the intermolecular stretching excitations not included in the 9D bending calculations herein. We expect that work currently in progress in our group, involving calculations that include all 12 intermolecular degrees of freedom of the trimer, will directly address this issue.

It is also notable in Figure 5a that relative intensities in the computed and observed spectra are not always in agreement. There are two likely sources for this. First, the approximation that we have made concerning the nature of the trimer’s dipole operator (see Section 2.9) may be at fault. In future work we will investigate the effect of including induced-dipole terms in the dipole operator. Second, the relative intensities in the He-nanodroplet mass-depletion spectrum may not match those in the corresponding absorption spectrum given that the former is an action spectrum,^25^ the signal of which depends on more than just the probability of photon absorption.

Apart from the nanodroplet spectrum, a second interesting comparison of our calculated spectra with experimental results can be made. The Saykally group has reported supersonic-beam absorption spectra in the 510 to 525 cm^–1^ spectral region.^31^ Their observation of four parallel bands at 517.2, 517.5, 523.9, and 525.3 cm^–1^ has been tentatively interpreted by them as evidence for very large (several cm^–1^) bifurcation tunneling splittings associated with the excitation of an out-of-plane librational mode of the trimer. Our computed results corresponding to this spectral region lend some support for this view, with a twist. In particular, as shown in Figure 6, we find a cluster of parallel bands near 514 cm^–1^ and a second cluster near 525 cm^–1^. The former can be readily assigned by wave function analysis to transitions from the  ground-state *G*_6_ level to a single  excited-state level. Similarly, the structure near 525 cm^–1^ corresponds to transitions from the ground-state level to a second, different  level. The structure of each cluster indicates very substantial bifurcation-tunneling splittings, as the 515 cm^–1^ features span about 0.6 cm^–1^, and the 525 cm^–1^ features span about 1.9 cm^–1^. In short, the calculated results support an interpetation whereby the observed ∼517 cm^–1^ and ∼525 cm^–1^ features arise from excitations to different  levels, which are indeed characterized by cm^–1^-magnitude bifurcation-tunneling splittings. As to the nature of these excited levels, we anticipate having more to add in an upcoming study focused on the characterization of the bend eigenstates.

One last feature of the calculated spectra is worthy of note. Namely, we find evidence for the violation of *G*_6_ dipole selection rules due to Fermi-resonance interactions between nearby states of the same *T* symmetry. For example, level transitions of the form  are not allowed under *G*_6_ selection rules, whereas  transitions are. And, our calculations do indeed show that such forbidden transitions have zero intensity when the excited  level is energetically isolated. However, when it is close in energy to an  level, the  component states of the former (e.g., see Figure 4) can couple effectively with those of the latter, as can the corresponding  states of the two levels. Via such coupling, states of the  level borrow perpendicular-band absorption intensity from those of the  level. A completely analogous situation obtains when an excited  level (which is *G*_6_-forbidden in a transition from the ground-state  level) is in proximity with an excited  level (which is *G*_6_-allowed in a transition from the ground state). In that case  components from the two levels can be couple strongly, as can  components, and states of the  level can borrow parallel-band absorption intensity from the  level.

We see two clear examples of intensity borrowing in our calculated spectra, both of which correspond to perpendicular bands. In one case an  and an  level are on top of each other at  cm^–1^, and the  states of the former borrow about one-quarter of the absorption intensity of the latter in transitions from the ground-state  components. In the second case, an  level is on top of an  level at  cm^–1^. The intensity borrowing in this case amounts to about one-sixth of the total *T*-to-*T* intensity in transitions from the ground-state level.

Notably, the bifurcation-tunneling splitting pattern for the bands of a level that gains intensity by the borrowing mechanism will be anomalous because the *A* states of the “dark” level cannot participate in the borrowing: There are no *A* states of common symmetry between the levels and, hence, no possibility of Fermi-resonance interaction. The *A* states of the dark level therefore do not contribute to the spectrum, unlike those of a regular allowed level.

## 4 Conclusions

We have presented the computational methodology for rigorous 9D quantum calculations of the intermolecular bending states of the water trimer. In this approach, the water monomers are assumed to be rigid, with their cm’s forming an equilateral triangle, and the intermonomer cm-to-cm distance is set to the expectation value of the ground state of the reduced-dimension 3D intermonomer stretching Hamiltonian. The remaining nine strongly coupled, highly anharmonic large-amplitude intermolecular bending (angular) DOFs are accounted for in the 9D bend Hamiltonian of the three fully coupled 3D rigid-water rotors. Solving for its 9D intermolecular eigenstates serves a 2-fold purpose. First, these eigenstates encompass excited librational vibrations together with their torsional and bifurcation tunneling splittings. Consequently, their accurate calculation constitutes the most rigorous, high-dimensional quantum treatment to date of the intermolecular vibrational states of the water trimer and the tunneling splittings in its excited states. Second, select low-energy 9D bend eigenstates, together with a certain number of lowest-energy 3D intermolecular stretching (or frame) eigenstates of the 3D frame Hamiltonian, comprise the final 12D product contracted basis for diagonalizing the full 12D intermolecular (bend + frame) vibrational Hamiltonian of the water trimer in the rigid-monomer approximation. Such calculations are ongoing in our group. In both the 9D calculations in this work and the 12D calculations under way, the high-quality 2 + 3-body PES of the water trimer by Zhang et al.^29^ is employed.

Calculating accurate eigenstates of the bend Hamiltonian having nine coupled large-amplitude intermolecular DOFs presents serious challenges. The high dimensionality of the problem demands that the final basis for the diagonalization of this 9D Hamiltonian is made maximally compact and efficient without sacrificing accuracy, in order to make the calculations feasible in a reasonable time. To achieve this, the basis needs to incorporate as much of the dynamics of the trimer as possible. This goal is accomplished by means of an elaborate computational scheme which fully exploits the *G*_48_ molecular symmetry group of the water trimer. Its central element are two cycles of generating contracted basis functions. In the first, 3D hindered-rotor states are obtained as the (3D) eigenstates of the rotating rigid-water monomer (its cm fixed) experiencing the potential of the two neighboring water monomers in the arrangement close to the equilibrium geometry of the water trimer. In the next step, a primitive basis of trilinear products of the hindered-rotor functions is used to diagonalize the 9D bend Hamiltonian, denoted as , for the potential that includes only the 2-body interaction terms and the 2-body approximation of the 3-body interaction term. In the final step, the full 9D bend Hamiltonian , whose potental includes both 2- and 3-body terms, is diagonalized in the truncated basis of the eigenvectors of  giving the desired 9D bending eigenstates.

The simulated low-frequency spectrum of the water trimer computed in this work using the 9D eigenstates of  shows remarkable agreement with the experimental FIR spectrum of the trimer in He nanodroplets^25^ over its entire range, both visually and in terms of the excellent match between the individual computed and measured spectral peaks. The fact that this is achieved although the present treatment does not include the intermolecular stretching vibrations of the trimer leads to the conclusion that most peaks in the measured FIR spectrum correspond to the intermolecular bending vibrations of the trimer, and that their coupling with the intermolecular stretching modes is weak. At a finer level of detail, the ground state torsional tunneling splittings computed in 9D in this work agree extremely well with experimental results from ref (43) and the full-dimensional PIMD calculations.^29^ On the other hand, the agreement of our 9D calculated bifurcation tunneling splittings with the spectroscopic data^43^ is only semiquantitative. Finally, the comparison between the present 9D calculations and the spectroscopic data for the water trimer points to the high quality of the *ab initio* IPES of Zhang et al.,^29^ at least in the 9D subspace of the bending vibrations probed in this work.

It is clear that the methodology for rigorous quantum 9D calculations of the bending eigenstates of the water trimer introduced in this work is already capable of providing a description of excited intermolecular trimer vibrations, and the associated tunneling splittings, with an unprecedented scope and level of accuracy and detail. This also gives us the tool to test the quality of the intermolecular PESs of water trimer far more comprehensively than has been possible previously.

Two immediate tasks remain for the near future. One of them is the analysis and assignment of the 9D bending eigenstates calculated in this work. This problem is very challenging, due to the high dimensionality of the eigenstates, their large-amplitude character, and strong coupling among the DOFs involved. But, it is important to gain understanding of the nature of the bending excitations, especially since they figure so prominently in the measured and calculated FIR spectra of the water trimer. The second task is extending the current 9D methodology to 12D, by including rigorously the intermolecular stretching vibrations and their coupling to the bending DOFs. This will enable a complete, fully coupled 12D quantum treatment of the intermolecular vibrations and tunneling splittings of the water trimer (for rigid monomers), as well as simulating the trimer FIR spectrum in 12D. Such calculations are nearing completion; the methodology employed and the 12D results will be reported in the near future.

## Supporting Information Available

Corrected expressions for kinetic-energy operators, details of the calculation of hindered-rotor states, proof that the , , and  operators are block-diagonal with respect to the *T* subirreps, symmetry selection rules for two-body matrix elements, symmetry characteristics of the and  quantities, monomer-permutation symmetry of  matrix elements, the exploitation of symmetry in the calculation of the  matrix elements, summary of results from the diagonalization of , and summary of results from the diagonalization of  (PDF)
