# Supplementary material for: Intermolecular Bending States and Tunneling Splittings of Water Trimer from Rigorous 9D Quantum Calculations: I. Methodology, Energy Levels, and Low-Frequency Spectrum
Source: J Phys Chem A. 2024 Sep 16;128(38):8170–89. doi: 10.1021/acs.jpca.4c05045 (PMC11440611; doi:10.1021/acs.jpca.4c05045)
Supplement: Supplementary file 1 — jp4c05045_si_001.pdf [file jp4c05045_si_001.pdf]

Supplementary Information for: Intermolecular Bending States and Tunneling Splittings of Water Trimer from Rigorous 9D Quantum Calculations: I. Methodology, Energy Levels, and Low-Frequency Spectrum

Peter M. Felker,[1, a] Irén Simkó,[2, 3, b] and Zlatko Bačić[2, 3, 4, c]

[1] *Department of Chemistry and Biochemistry, University of California, Los Angeles, California 90095-1569, USA*

[2] *Department of Chemistry, New York University, New York, New York 10003, USA*

[3] *Simons Center for Computational Physical Chemistry at New York University, New York, New York 10003, USA*

[4] *NYU-ECNU Center for Computational Chemistry at NYU Shanghai, 3663 Zhongshan Road North, Shanghai, 200062, China*

(Dated: 26 August 2024)

[a] Electronic mail: felker@chem.ucla.edu

[b] Electronic mail: is2873@nyu.edu

[c] Electronic mail: zlatko.bacic@nyu.edu

S1. CORRECTED EXPRESSIONS FOR THE OPERATORS $\hat{K}_F(R)$ AND $V'(R)$

There are misprint errors in the expressions for the kinetic-energy operators $\hat{K}_F(R)$ and $V'(R)$ in Refs. 34 and 38 cited in the main text. The following, which restate Eqs. (5) and (6) of Ref. 34, are the correct expressions:

$$\hat{K}_F(R) \equiv \sum_{k=1}^{3}\left[-\frac{1}{2\mu_k}\frac{\partial^2}{\partial R_k^2} - \frac{\cos\alpha_k}{2R_1R_2R_3}\left(\frac{R_l^2}{M_l}+\frac{R_m^2}{M_m}\right)\frac{\partial}{\partial R_k}\right] \tag{S1}$$

$$-\left[\frac{\cos\alpha_1}{M_1}\frac{\partial^2}{\partial R_2\partial R_3}+\frac{\cos\alpha_1}{M_1}\frac{\partial^2}{\partial R_2\partial R_3}+\frac{\cos\alpha_3}{M_3}\frac{\partial^2}{\partial R_1\partial R_2}\right] \tag{S2}$$

and

$$\hat{V}'(R) \equiv \frac{\frac{R_1^4}{M_1}+\frac{R_2^4}{M_2}+\frac{R_3^4}{M_3}}{8R_1^2R_2^2R_3^2} \tag{S3}$$

S2. DETAILS OF THE CALCULATION OF THE HINDERED-ROTOR STATES

We used the Chebyshev version[1] of filter diagonalization[2] to solve for the hindered-rotor states in a basis set consisting of normalized, symmetric-top rotational eigenstates (see Eq. (19) of the main text). This algorithm requires the iterative operation of the pertinent Hamiltonian ($\hat{H}_A^{(n)}$, see Eqs. (13) and (16) of the main text) on an initially random state vector. In respect to the kinetic-energy portion of $\hat{H}_A^{(n)}$ such operation is effected by matrix-vector multiplication, the matrix elements of $\hat{T}_A$ in the symmetric-top basis being easily available analytically. As to the operation with $V_A^{(n)}(\omega_A)$, we transform the state vector to a 3D grid representation $(\phi_{A,t}, \cos(\theta_A)_u, \chi_{A,v})$, $(t = 1,\ldots,N_\phi)$, $(u = 1,\ldots,N_\theta)$, $(v = 1,\ldots,N_\chi)$, where the $\phi_{A,t}$ and $\chi_{A,v}$ constitute 1D Fourier grids and the $\cos(\theta_A)_u$ are Gauss-Legendre quadrature points. We then multiply the state vector at each grid point by the value of $V_A^{(n)}(\omega_A)$ at that grid point. Finally, we transform the result back to the basis-state representation. As mentioned in the main text, the hindered-rotor eigenvectors ultimately obtained are given as expansions over the symmetric-top-eigenfunction basis states

$$|\alpha(\omega_I)\rangle = \sum_{j_I=0}^{j_{\max}}\sum_{k_I=-j_I}^{j_I}\sum_{m_I=-j_I}^{j_I}|j_I,k_I,m_I\rangle\langle j_I,k_I,m_I|\alpha\rangle \tag{S4}$$

S3. THE MATRIX ELEMENTS OF $\hat{H}^{(2B)}_{\text{bend}}$ AND $\hat{H}_{\text{bend}}$ BETWEEN STATES BELONGING TO DIFFERENT T SUBIRREPS EQUAL ZERO

Consider the matrix element $\langle\Phi_{T^+_{g,a}}|\hat{H}^{(2B)}_{\text{bend}}|\Phi'_{T^+_{g,b}}\rangle$, where $|\Phi_{T^+_{g,a}}\rangle$ is a 9D basis state that transforms as the $T^+_{g,a}$ sub-irrep of T^+_g and $|\Phi'_{T^+_{g,b}}\rangle$ is a basis state that transforms as the $T^+_{g,b}$ sub-irrep of that same irrep.

$$\begin{aligned}\langle\Phi_{T^+_{g,a}}|\hat{H}^{(2B)}_{\text{bend}}|\Phi'_{T^+_{g,b}}\rangle &= \langle\Phi_{T^+_{g,a}}|(12)^{-1}[(12)\hat{H}^{(2B)}_{\text{bend}}(12)^{-1}](12)|\Phi'_{T^+_{g,b}}\rangle \\ &= -\langle\Phi_{T^+_{g,a}}|\hat{H}^{(2B)}_{\text{bend}}|\Phi'_{T^+_{g,b}}\rangle = 0\end{aligned} \tag{S5}$$

where we have used the invariance of $\hat{H}^{(2B)}_{\text{bend}}$ with respect to (12) and the fact that $(12)|\Phi_{T^+_{g,a}}\rangle = |\Phi_{T^+_{g,a}}\rangle$ and $(12)|\Phi'_{T^+_{g,b}}\rangle = -|\Phi'_{T^+_{g,b}}\rangle$. Similar relations involving the other monomer H-exchange operators [(i.e., (34) and (56)] can be easily obtained for all $\hat{H}^{(2B)}_{\text{bend}}$ matrix elements connecting states of different T sub-irreps. Since $\hat{H}_{\text{bend}}$ is also invariant with respect to all the monomer H-exchange operators, its matrix elements connecting different T sub-irreps are also equal to zero. The upshot is that the matrices of $\hat{H}^{(2B)}_{\text{bend}}$ and $\hat{H}_{\text{bend}}$ are both block diagonal with respect to the T sub-irreps.

S4. SYMMETRY SELECTION RULES FOR THE TWO-BODY MATRIX ELEMENTS $\langle\alpha',\beta'|\hat{T}_{I,J}|\alpha,\beta\rangle$ AND $\langle\alpha',\beta'|V^{(2B)}_{\text{bend}}(\omega_I,\omega_J)|\alpha,\beta\rangle$

The two-body operators $\hat{T}_{I,J}$ and $\hat{V}^{(2B)}_{\text{bend}}(\omega_I,\omega_J)$ are both invariant with respect to E^* and with respect to the H-exchange operators associated with monomers I and J. Hence,

$$\langle\alpha',\beta'|\hat{O}_{I,J}|\alpha,\beta\rangle = \langle\alpha',\beta'|\hat{R}^{-1}|[\hat{R}\hat{O}_{I,J}\hat{R}^{-1}]|\hat{R}|\alpha,\beta\rangle = [\langle\alpha',\beta'|\hat{R}^{-1}]\hat{O}_{I,J}[\hat{R}|\alpha,\beta\rangle] \tag{S6}$$

where $\hat{O}$ is either $\hat{T}_{I,J}$ or $V^{(2B)}_{\text{bend}}$, and $\hat{R}$ is one of the aforementioned symmetry operators. With $\hat{R} = E^*$ Eq. (S6) becomes

$$\langle\alpha',\beta'|\hat{O}_{I,J}|\alpha,\beta\rangle = p_{\alpha'}p_{\beta'}p_\alpha p_\beta\langle\alpha',\beta'|\hat{O}_{I,J}|\alpha,\beta\rangle \tag{S7}$$

since $E^*|\alpha,\beta\rangle = p_\alpha p_\beta|\alpha,\beta\rangle$, where $p_\alpha = \pm 1$ and $p_\beta = \pm 1$. From Eq. (S7) it follows that the matrix elements equal zero unless $p_{\alpha'}p_{\beta'}p_\alpha p_\beta = +1$.

Similarly, with $\hat{R}$ the H-exchange operator associated with monomer I, one has

$$\langle\alpha',\beta'|\hat{O}_{I,J}|\alpha,\beta\rangle = q_{\alpha'}q_\alpha\langle\alpha',\beta'|\hat{O}_{I,J}|\alpha,\beta\rangle \tag{S8}$$

and when $\hat{R}$ is the H-exchange operator associated with monomer J

$$\langle\alpha',\beta'|\hat{O}_{I,J}|\alpha,\beta\rangle = q_{\beta'}q_{\beta}\langle\alpha',\beta'|\hat{O}_{I,J}|\alpha,\beta\rangle \tag{S9}$$

where the H-exchange eigenvalues q_α, etc. each equal +1 or −1. From Eqs. (S8) and (S9) it follows that the matrix elements are zero unless both $q_\alpha q_{\alpha'}$ and $q_\beta q_{\beta'}$ equal +1.

S5. PROPERTIES OF THE $V^{(2B)}_{\lambda_I,\lambda_J}$ AND $T^{\lambda_I}_{\alpha',\alpha}$ DUE TO SYMMETRY

A. Effects of H-exchange symmetry

Since $V^{(2B)}_{\lambda_A,\lambda_B}$ is a scalar number, it is invariant to H-nuclei-exchange on either of the two monomers. This along with the defining equation for the $V^{(2B)}_{\lambda_A,\lambda_B}$ yields

$$\begin{aligned}(12)V^{(2B)}_{\lambda_A,\lambda_B} = V^{(2B)}_{\lambda_A,\lambda_B} &= \int (12)V^{(2B)}_{\text{bend}}(\omega_A,\omega_B)(12)^{-1}(12)\left[\tilde{D}^{j_A}_{m_A,k_A}(\omega_A)\tilde{D}^{j_B}_{m_B,k_B}(\omega_B)\right]d\omega_A d\omega_B \\ &= \int V^{(2B)}_{\text{bend}}(\omega_A,\omega_B)\left[(-1)^{k_A}\tilde{D}^{j_A}_{m_A,k_A}(\omega_A)\tilde{D}^{j_B}_{m_B,k_B}(\omega_B)\right]d\omega_A d\omega_B \\ &= (-1)^{k_A}V^{(2B)}_{\lambda_A,\lambda_B}\end{aligned} \tag{S10}$$

where we have used the invariance of $V^{(2B)}_{\text{bend}}(\omega_A,\omega_B)$ with respect to (12):

$$(12)V^{(2B)}_{\text{bend}}(\omega_A,\omega_B)(12)^{-1} = V^{(2B)}_{\text{bend}}(\omega_A,\omega_B). \tag{S11}$$

Similarly, $V^{(2B)}_{\lambda_A,\lambda_B} = (-1)^{k_B}V^{(2B)}_{\lambda_A,\lambda_B}$. Thus, only $V^{(2B)}_{\lambda_A,\lambda_B}$ expansion coefficients corresponding to even values of both k_A and k_B are nonzero. Completely analogous results apply to the $V^{(2B)}_{\lambda_B,\lambda_C}$ and $V^{(2B)}_{\lambda_C,\lambda_A}$ expansion coefficients.

B. Effects of E^* symmetry

Similarly, $V^{(2B)}_{\lambda_I,\lambda_J}$ and $V^{(2B)}_{\text{bend}}(\omega_I,\omega_J)$ are invariant to E^*. Thus,

$$\begin{aligned}E^*V^{(2B)}_{\lambda_I,\lambda_J} = V_{\lambda_A,\lambda_B} &= \int [E^*V^{(2B)}_{\text{bend}}(\omega_A,\omega_B)E^*]E^*\left[\tilde{D}^{j_A}_{m_A,k_A}(\omega_A)\tilde{D}^{j_B}_{m_B,k_B}(\omega_B)\right]d\omega_A d\omega_B \\ &= \int V^{(2B)}_{\text{bend}}(\omega_A,\omega_B)E^*\left[\tilde{D}^{j_A}_{m_A,k_A}(\omega_A)\tilde{D}^{j_B}_{m_B,k_B}(\omega_B)\right]d\omega_A d\omega_B\end{aligned} \tag{S12}$$

where the second equality follows from the invariance of $V^{(2B)}_{\text{bend}}$ to E^*. Now,

$$E^*\left[\tilde{D}^{j_A}_{m_A,k_A}(\omega_A)\tilde{D}^{j_B}_{m_B,k_B}(\omega_B)\right] = (-1)^{j_A+m_A+j_B+m_B} D^{j_A}_{m_A,\bar{k}_A}(\omega_A)\tilde{D}^{j_B}_{m_B,\bar{k}_B}(\omega_B) \tag{S13}$$

where $\bar{k} \equiv -k$. Equation (S13) along with Eq. (S12) yields

$$V^{(2B)}_{\lambda_A,\lambda_B} = (-1)^{J+M} V^{(2B)}_{\bar{\lambda}_A,\bar{\lambda}_B} \tag{S14}$$

where $\bar{\lambda}_I \equiv (j_I, -k_I, m_I)$, $J \equiv j_A + j_B$ and $M \equiv m_A + m_B$.

$V^{(2B)}_{\lambda_I,\lambda_J}$ enters into the expression for the matrix elements of $V^{(2B)}_{\text{bend}}$ in the $|\alpha,\beta,\gamma\rangle$ basis along with quantities of the form

$$T^{\lambda_I}_{\alpha',\alpha}T^{\lambda_J}_{\beta',\beta} = \langle\alpha'|[\tilde{D}^{(j_I} m_I, k_I(\omega_I)]^*|\alpha\rangle\langle\beta'|[\tilde{D}^{(j_J} m_J, k_J(\omega_J)]^*|\beta\rangle \tag{S15}$$

Considering the effect of E^* on $T^{\lambda_I}_{\alpha',\alpha}$ one finds

$$\begin{aligned} T^{\lambda_I}_{\alpha',\alpha} &= \langle\alpha'|E^*E^*[\tilde{D}^{(j_I)}_{m_I,k_I}(\omega_I)]^*E^*E^*|\alpha\rangle = p_{\alpha'}p_\alpha\langle\alpha'|E^*[\tilde{D}^{(j_I)}_{m_I,k_I}(\omega_I)]^*E^*|\alpha\rangle \\ &= p_{\alpha'}p_\alpha(-1)^{j_I+m_I}\langle\alpha'|[\tilde{D}^{(j_I)}_{m_I,-k_I}(\omega_I)]^*|\alpha\rangle = (-1)^{j_I+m_I}p_{\alpha'}p_\alpha T^{\bar{\lambda}_I}_{\alpha',\alpha} \end{aligned} \tag{S16}$$

where $\bar{\lambda}_I \equiv (j_I, -k_I, m_i)$ and p_α and $p_{\alpha'}$ are, respectively, the eigenvalues of $|\alpha\rangle$ and $|\alpha'\rangle$ with respect to E^*. Similarly, one finds

$$T^{\lambda_J}_{\beta',\beta} = (-1)^{j_J+m_J}p_{\beta'}p_\beta T^{\bar{\lambda}_J}_{\beta',\beta} \tag{S17}$$

So,

$$T^{\lambda_I}_{\alpha',\alpha}T^{\lambda_J}_{\beta',\beta} = (-1)^{j_I+j_J+m_I+m_J}p_{\alpha'}p_\alpha p_{\beta'}p_\beta T^{\bar{\lambda}_I}_{\alpha',\alpha}T^{\bar{\lambda}_J}_{\beta',\beta} \tag{S18}$$

Given that

$$V^{(2B)}_{\lambda_I,\lambda_J} = (-1)^{j_I+j_J+m_I+m_J}V^{(2B)}_{\bar{\lambda}_I,\bar{\lambda}_J} \tag{S19}$$

one has

$$V^{(2B)}_{\lambda_I,\lambda_J}T^{\lambda_I}_{\alpha',\alpha}T^{\lambda_J}_{\beta',\beta} = p_{\alpha'}p_\alpha p_{\beta'}p_\beta V^{(2B)}_{\bar{\lambda}_I,\bar{\lambda}_J}T^{\bar{\lambda}_I}_{\alpha',\alpha}T^{\bar{\lambda}_J} \tag{S20}$$

Since the $\langle\alpha',\beta'|V^{(2B)}_{\text{bend}}|\alpha,\beta\rangle$ are only nonzero if $p_{\alpha'}p_\alpha p_{\beta'}p_\beta = +1$, one effectively has

$$V^{(2B)}_{\lambda_I,\lambda_J}T^{\lambda_I}_{\alpha',\alpha}T^{\lambda_J}_{\beta',\beta} = V^{(2B)}_{\bar{\lambda}_I,\bar{\lambda}_J}T^{\bar{\lambda}_I}_{\alpha',\alpha}T^{\bar{\lambda}_J}_{\beta',\beta} \tag{S21}$$

Equation (S21) allows for the reduction by about a factor of two in the cost of computing the $\langle\alpha',\beta'|V^{(2B)}_{\text{bend}}|\alpha,\beta\rangle$ matrix elements.

S6. MONOMER-PERMUTATION SYMMETRY OF $\hat{H}_{\text{bend}}^{(2B)}$ MATRIX ELEMENTS IN THE HINDERED-ROTOR BASIS

Here, we prove that for Γ equal to one of the A irreps

$$\langle \alpha, \beta, \gamma | \hat{T}_A | \psi_\Gamma \rangle = \delta \langle \beta, \gamma, \alpha | \hat{T}_C | \psi_\Gamma \rangle = \delta^* \langle \gamma, \alpha, \beta | \hat{T}_B | \psi_\Gamma \rangle \tag{S22}$$

and

$$\langle \alpha, \beta, \gamma | (\hat{T}_{A,B} + V_{A,B}) | \psi_\Gamma \rangle = \delta \langle \beta, \gamma, \alpha | (\hat{T}_{C,A} + V_{C,A}) | \psi_\Gamma \rangle = \delta^* \langle \gamma, \alpha, \beta | (\hat{T}_{B,C} + V_{B,C}) | \psi_\Gamma \rangle \tag{S23}$$

where $\delta = (+1, e^{i2\pi/3}, e^{-i2\pi/3})$ for $\Gamma = (A^{\pm}_{1g/1u}, A^{\pm}_{2g/2u}, A^{\pm}_{3g/3u})$.

We use

$$(ABC)(135)(246) | \psi_\Gamma \rangle = \delta | \psi_\Gamma \rangle \tag{S24}$$

and

$$(ACB)(153)(264) | \psi_\Gamma \rangle = \delta^* | \psi_\Gamma \rangle, \tag{S25}$$

where $|\psi_\Gamma\rangle$ is a state function that belongs to irrep Γ. We also use

$$(ABC)(135)(246) | \alpha, \beta, \gamma \rangle = | \beta, \gamma, \alpha \rangle \quad \text{and} \quad (ACB)(153)(264) | \alpha, \beta, \gamma \rangle = | \gamma, \alpha, \beta \rangle \tag{S26}$$

together with

$$\hat{O} \hat{T}_A \hat{O}^{-1} = \hat{T}_C \quad \text{and} \quad \hat{O}^{-1} \hat{T}_A \hat{O} = \hat{T}_B \tag{S27}$$

and

$$\hat{O} (\hat{T}_{A,B} + V_{A,B}) \hat{O}^{-1} = \hat{T}_{C,A} + V_{C,A} \quad \text{and} \quad \hat{O}^{-1} (\hat{T}_{A,B} + V_{A,B}) \hat{O} = \hat{T}_{B,C} + V_{B,C} \tag{S28}$$

where $\hat{O} \equiv (ABC)(135)(246)$ and, thus, $\hat{O}^{-1} = (ACB)(153)(264)$.

The proofs follow directly from these relations when applied to

$$\langle \alpha, \beta, \gamma | \hat{T}_A | \psi_\Gamma \rangle = \langle \alpha, \beta, \gamma | \hat{O}^{-1} [\hat{O} \hat{T}_A \hat{O}^{-1}] \hat{O} | \psi_\Gamma \rangle = \langle \alpha, \beta, \gamma | \hat{O} [\hat{O}^{-1} \hat{T}_A \hat{O}] \hat{O}^{-1} | \psi_\Gamma \rangle \tag{S29}$$

and

$$\begin{aligned} \langle \alpha, \beta, \gamma | \hat{T}_{A,B} + V_{A,B} | \psi_\Gamma \rangle &= \langle \alpha, \beta, \gamma | \hat{O}^{-1} [\hat{O} (\hat{T}_{A,B} + V_{A,B}) \hat{O}^{-1}] \hat{O} | \psi_\Gamma \rangle \\ &= \langle \alpha, \beta, \gamma | \hat{O} [\hat{O}^{-1} (\hat{T}_{A,B} + V_{A,B}) \hat{O}] \hat{O}^{-1} | \psi_\Gamma \rangle \end{aligned} \tag{S30}$$

S7. THE REDUCTION OF COMPUTATIONAL EFFORT IN THE CALCULATION OF $V_{\rm bend}^{(3B)}$ MATRIX ELEMENTS BY THE EXPLOITATION OF SYMMETRY

The evaluation of the quantities

$$\langle\alpha',\beta',\gamma'|V_{\rm bend}^{(3B)}|\sigma_\Gamma\rangle = \sum_{\lambda_A,\lambda_B,\lambda_C} V_{\lambda_A,\lambda_B,\lambda_C}^{(3B)} \sum_{\alpha,\beta,\gamma} T_{\alpha',\alpha}^{\lambda_A} T_{\beta',\beta}^{\lambda_B} T_{\gamma',\gamma}^{\lambda_C} \langle\alpha,\beta,\gamma|\sigma_\Gamma\rangle \tag{S31}$$

is the costliest step in the evaluation of the matrix elements of $V_{\rm bend}^{(3B)}$ in the $|\sigma_\Gamma\rangle$ bases. By exploiting symmetry, one can reduce significantly the effort required to complete these calculations.

First, because the $V_{\lambda_A,\lambda_B,\lambda_C}^{(3B)}$ are only nonzero for even k_A, k_B, and k_C, the number of terms in the $\lambda_A,\lambda_B,\lambda_C$ is about a factor of eight smaller than if all possible values of the k_I were significant. This reduction arises from the trimer's monomer-H-exchange symmetries.

Moving on to examine the effects of E^* and monomer-permutation symmetry, we re-write Eq. (S31) as

$$\langle\alpha',\beta',\gamma'|V_{\rm bend}^{(3B)}|\sigma_\Gamma\rangle = \sum_{\lambda_A,\lambda_B,\lambda_C} V_{\lambda_A,\lambda_B,\lambda_C}^{(3B)} \langle\alpha'\beta'\gamma'|\tau^{\lambda_A,\lambda_B,\lambda_C}|\sigma_\Gamma\rangle \tag{S32}$$

where

$$\tau^{\lambda_A,\lambda_B,\lambda_C} \equiv T^{\lambda_A}(\omega_A)T^{\lambda_B}(\omega_B)T^{\lambda_C}(\omega_C) \equiv [\tilde{D}_{m_A,k_A}^{j_A}(\omega_A)\tilde{D}_{m_B,k_B}^{j_B}(\omega_B)\tilde{D}_{m_C,k_C}^{j_C}(\omega_C)]^* \tag{S33}$$

From the expression for the expansion coefficients

$$V_{\lambda_A,\lambda_B,\lambda_C}^{(3B)} = \int\int\int V_{\rm bend}^{(3B)}(\omega_A,\omega_B,\omega_C)\tilde{D}_{m_A,k_A}^{(j_A)}(\omega_A)\tilde{D}_{m_B,k_B}^{(j_B)}(\omega_B)\tilde{D}_{m_C,k_C}^{(j_C)}(\omega_C)d\omega_A d\omega_B d\omega_C \tag{S34}$$

it is straightforward to show that due to E^* symmetry

$$V_{\bar{\lambda}_A,\bar{\lambda}_B,\bar{\lambda}_C}^{(3B)} = (-1)^{J+M} V_{\lambda_A,\lambda_B,\lambda_C}^{(3B)} \tag{S35}$$

where $\bar{\lambda}_I \equiv (j_I,-k_I,m_I)$ and $J+M \equiv j_A+j_B+j_C+m_A+m_B+m_C$, and that due to $(ABC)(135)(246)$ and $(ACB)(264)(153)$ symmetry

$$V_{\lambda_A,\lambda_B,\lambda_C}^{(3B)} = V_{\lambda_B,\lambda_C,\lambda_A}^{(3B)} = V_{\lambda_C,\lambda_A,\lambda_B}^{(3B)} \tag{S36}$$

Consider next the effects of these symmetries on the $\langle\alpha'\beta'\gamma'|\tau^{\lambda_A,\lambda_B,\lambda_C}|\sigma_\Gamma\rangle$. In respect to E^* one has

$$\begin{aligned}\langle\alpha'\beta'\gamma'|\tau^{\lambda_A,\lambda_B,\lambda_C}|\sigma_\Gamma\rangle &= \langle\alpha'\beta'\gamma'|(E^*)^{-1}\left[E^*\tau^{\lambda_A,\lambda_B,\lambda_C}\hat{(}E^*)^{-1}\right]E^*|\sigma_\Gamma\rangle \\ &= (-1)^{J+M}\langle\alpha'\beta'\gamma'|\tau^{\bar{\lambda}_A,\bar{\lambda}_B,\bar{\lambda}_C}|\sigma_\Gamma\rangle\end{aligned} \tag{S37}$$

where we have used the facts that $|\sigma_\Gamma\rangle$ and $|\alpha'\beta'\gamma'\rangle$ are both eigenfunctions of E^* with the same eigenvalue (i.e., ±1) and that $E^*\tilde{D}^{(j)}_{m,k}(\omega)E^* = (-1)^{j+m}\tilde{D}^{(j)}_{m,-k}(\omega)$.

In respect to $(ABC)(135)(246)$ and $(ACB)(153)(264)$ one has for Γ equal to one of the A irreps

$$\langle\alpha',\beta',\gamma'|\tau^{\lambda_A,\lambda_B,\lambda_C}|\sigma_\Gamma\rangle = \langle\alpha',\beta',\gamma'|\hat{O}^{-1}\left[\hat{O}[\tau^{\lambda_A,\lambda_B,\lambda_C}]\hat{O}^{-1}\right]\hat{O}|\sigma_\Gamma\rangle = \delta\langle\beta',\gamma',\alpha'|\tau^{\lambda_B,\lambda_C,\lambda_A}|\sigma_\Gamma\rangle \tag{S38}$$

when $\hat{O} = (ABC)(135)(246)$, and when $\hat{O} = (ACB)(153)(264)$

$$\langle\alpha',\beta',\gamma'|\tau^{\lambda_A,\lambda_B,\lambda_C}|\sigma_\Gamma\rangle = \delta^*\langle\gamma',\alpha',\beta'|\tau^{\lambda_C,\lambda_A,\lambda_B}|\sigma_\Gamma\rangle \tag{S39}$$

Given Eqs. (S38) and (S39) it is easy to show that

$$\begin{aligned}\langle\alpha',\beta',\gamma'|(\tau^{\lambda_A,\lambda_B,\lambda_C}+\tau^{\lambda_B,\lambda_C,\lambda_A} &+ \tau^{\lambda_C,\lambda_A,\lambda_B})|\sigma_\Gamma\rangle = \langle\alpha',\beta',\gamma'|\tau^{\lambda_A,\lambda_B,\lambda_C}|\sigma_\Gamma\rangle \\ &+ \delta\langle\beta',\gamma',\alpha'|\tau^{\lambda_A,\lambda_B,\lambda_C}|\sigma_\Gamma\rangle + \delta^*\langle\gamma',\alpha',\beta'|\tau^{\lambda_A,\lambda_B,\lambda_C}|\sigma_\Gamma\rangle\end{aligned} \tag{S40}$$

Putting Eqs. (S35) and (S37) together one has

$$\begin{aligned}V^{(3B)}_{\lambda_A,\lambda_B,\lambda_C}\langle\alpha',\beta',\gamma'|\tau^{\lambda_A,\lambda_B,\lambda_C}|\sigma_\Gamma\rangle &+ V^{(3B)}_{\bar{\lambda}_A,\bar{\lambda}_B,\bar{\lambda}_C}\langle\alpha',\beta',\gamma'|\tau^{\bar{\lambda}_A,\bar{\lambda}_B,\bar{\lambda}_C}|\sigma_\Gamma\rangle \\ &= 2V^{(3B)}_{\lambda_A,\lambda_B,\lambda_C}\langle\alpha',\beta',\gamma'|\tau^{\lambda_A,\lambda_B,\lambda_C}|\sigma_\Gamma\rangle\end{aligned} \tag{S41}$$

This allows for a reduction in the number of terms on the rhs of Eq. (S32) [or Eq. (S31)] by about a factor of two.

With Eqs. (S36) and (S40) one has

$$\begin{aligned} V^{(3B)}_{\lambda_A,\lambda_B,\lambda_C} &\langle \alpha', \beta', \gamma' | \tau^{\lambda_A,\lambda_B,\lambda_C} | \sigma_\Gamma \rangle + V^{(3B)}_{\lambda_B,\lambda_C,\lambda_A} \langle \alpha', \beta', \gamma' | \tau^{\lambda_B,\lambda_C,\lambda_A} | \sigma_\Gamma \rangle \\ &+ V^{(3B)}_{\lambda_C,\lambda_A,\lambda_B} \langle \alpha', \beta', \gamma' | \tau^{\lambda_C,\lambda_A,\lambda_B}) | \sigma_\Gamma \rangle = \\ &V^{(3B)}_{\lambda_A,\lambda_B,\lambda_C} \Big[\langle \alpha', \beta', \gamma' | \tau^{\lambda_A,\lambda_B,\lambda_C} | \sigma_\Gamma \rangle + \delta \langle \beta', \gamma', \alpha' | \tau^{\lambda_A,\lambda_B,\lambda_C} | \sigma_\Gamma \rangle \\ &+ \delta^* \langle \gamma', \alpha', \beta' | \tau^{\lambda_A,\lambda_B,\lambda_C} | \sigma_\Gamma \rangle \Big] \end{aligned} \tag{S42}$$

Equation (S42) implies that by computing $V^{(3B)}_{\lambda_A,\lambda_B,\lambda_C} \langle \alpha', \beta', \gamma' | \tau^{\lambda_A,\lambda_B,\lambda_C} | \sigma_\Gamma \rangle$ for all α', β', γ', one also has with trivial additional effort the quantities $V^{(3B)}_{\lambda_B,\lambda_C,\lambda_A} \langle \alpha', \beta', \gamma' | \tau^{\lambda_B,\lambda_C,\lambda_A} | \sigma_\Gamma \rangle$ and $V^{(3B)}_{\lambda_C,\lambda_A,\lambda_B} \langle \alpha', \beta', \gamma' | \tau^{\lambda_C,\lambda_A,\lambda_A} | \sigma_\Gamma \rangle$ for all α', β', γ'. Thus, for Γ equal to an A irrep, the cost of computing Eq. (S32) [or Eq. (S31)] is reduced by about a factor of three.

In sum, the G_{48} symmetry of water trimer allows for a cost reduction of about 48 for A irreps and about 16 for T sub-irreps in the calculation of matrix elements of $V^{(3B)}_{\text{bend}}$ in the $|\sigma_\Gamma\rangle$ bases.

S8. SUMMARY OF RESULTS FROM THE DIAGONALIZATION OF $\hat{H}^{(2B)}_{\text{bend}}$

Following are results pertaining to the 30/80 lowest-energy $\hat{H}^{(2B)}_{\text{bend}}$ eigenstates computed for each of the A/T G_{48} irreps. In each Table ΔE is the energy of the state relative to that of the $\hat{H}^{(2B)}_{\text{bend}}$ ground state ($A^+_{1g}(1)$) at -4016.162 cm^{-1}. BSN$_{100}$ is the sum of the basis-state norms corresponding to the 100 symmetry-adapted hindered-rotor basis states that contribute the most to each eigenstate.

TABLE S1. Computed $\hat{H}_{\text{bend}}^{(2B)}$ eigenstates for the A_{1g}^{+} and A_{1u}^{+} irreps.

	A_{1g}^{+}		A_{1u}^{+}	
N	ΔE/ cm^{-1}	BSN$_{100}$	ΔE/ cm^{-1}	BSN$_{100}$
1	0.000	0.9912	0.013	0.9916
2	145.486	0.9798	145.481	0.9803
3	207.678	0.9769	207.687	0.9773
4	271.513	0.9555	271.686	0.9554
5	277.083	0.9553	277.145	0.9560
6	311.151	0.9499	311.561	0.9495
7	320.554	0.9548	320.391	0.9542
8	344.902	0.9512	344.903	0.9512
9	378.106	0.9417	378.938	0.9420
10	406.225	0.9308	406.235	0.9309
11	419.412	0.9245	419.311	0.9258
12	426.352	0.9245	426.687	0.9249
13	444.177	0.9253	444.180	0.9243
14	447.067	0.9407	447.931	0.9395
15	462.841	0.9229	463.004	0.9218
16	476.616	0.9358	476.633	0.9364
17	490.498	0.8921	489.401	0.8926
18	494.155	0.9053	494.954	0.9098
19	509.692	0.8987	511.141	0.8952
20	517.166	0.8997	517.062	0.8986
21	522.296	0.8974	525.411	0.8937
22	530.847	0.9060	530.588	0.9145
23	539.081	0.8859	542.182	0.8944
24	543.462	0.8831	543.049	0.8785
25	548.354	0.8972	549.459	0.8869
26	552.388	0.8938	556.350	0.8940
27	561.731	0.8701	562.495	0.8747
28	570.564	0.8892	571.005	0.8902
29	581.072	0.8855	581.105	0.8868
30	588.310	0.8806	588.099	0.8829

TABLE S2. Computed $\hat{H}_{\text{bend}}^{(2B)}$ eigenstates for the $A_{2g/3g}^{-}$ and $A_{2u/3u}^{-}$ irreps.

	$A_{2g/3g}^{-}$		$A_{2u/3u}^{-}$	
N	$\Delta E/$ cm^{-1}	BSN$_{100}$	$\Delta E/$ cm^{-1}	BSN$_{100}$
1	24.010	0.9864	24.007	0.9865
2	193.687	0.9702	193.692	0.9704
3	215.272	0.9614	215.300	0.9613
4	271.126	0.9579	270.957	0.9579
5	301.790	0.9550	301.345	0.9549
6	322.605	0.9365	322.711	0.9371
7	344.162	0.9350	344.258	0.9337
8	376.298	0.9283	376.381	0.9283
9	383.556	0.9359	383.016	0.9345
10	414.538	0.9178	414.684	0.9187
11	422.830	0.9199	423.034	0.9218
12	434.831	0.9187	434.688	0.9207
13	445.153	0.9180	445.102	0.9181
14	455.894	0.9014	455.703	0.9015
15	473.827	0.9015	473.515	0.9024
16	479.813	0.9165	479.636	0.9171
17	489.966	0.9091	489.046	0.9028
18	496.355	0.9071	495.768	0.9076
19	510.433	0.8977	506.526	0.8993
20	522.114	0.8837	520.810	0.8863
21	530.465	0.8794	530.486	0.8776
22	545.186	0.8743	544.368	0.8740
23	548.809	0.8745	549.328	0.8738
24	552.615	0.8827	552.148	0.8834
25	562.106	0.8755	561.066	0.8706
26	570.164	0.8623	568.300	0.8687
27	581.627	0.8726	580.265	0.8601
28	588.337	0.8488	588.616	0.8577
29	590.874	0.8562	595.572	0.8550
30	597.561	0.8607	597.645	0.8519

TABLE S3. Computed $\hat{H}_{\text{bend}}^{(2B)}$ eigenstates for the $A^+_{2g/3g}$ and $A^+_{2u/3u}$ irreps.

	$A^+_{2g/3g}$		$A^+_{2u/3u}$	
N	$\Delta E/$ cm^{-1}	BSN$_{100}$	$\Delta E/$ cm^{-1}	BSN$_{100}$
1	68.336	0.9833	68.337	0.9834
2	150.498	0.9753	150.585	0.9761
3	229.590	0.9565	229.607	0.9567
4	276.074	0.9452	276.230	0.9441
5	306.849	0.9456	306.899	0.9450
6	319.194	0.9519	319.147	0.9525
7	345.904	0.9353	346.020	0.9366
8	364.130	0.9431	364.063	0.9438
9	378.835	0.9326	378.914	0.9330
10	397.765	0.9371	399.286	0.9360
11	422.596	0.9240	423.226	0.9230
12	440.939	0.9168	440.855	0.9171
13	454.927	0.9028	454.997	0.9023
14	461.039	0.8987	461.508	0.8991
15	474.724	0.9000	474.584	0.9000
16	490.119	0.9051	491.149	0.9038
17	493.698	0.9065	493.711	0.9083
18	509.977	0.8939	509.100	0.8951
19	518.292	0.9005	518.643	0.9001
20	523.411	0.8863	523.472	0.8846
21	533.610	0.8854	537.596	0.8769
22	541.859	0.8858	541.598	0.8842
23	547.707	0.8801	548.790	0.8910
24	552.298	0.8865	552.100	0.8813
25	557.742	0.8890	559.392	0.8816
26	565.591	0.8802	566.346	0.8858
27	574.127	0.8751	574.447	0.8779
28	579.808	0.8615	583.531	0.8632
29	586.481	0.8737	586.606	0.8631
30	594.948	0.8593	599.308	0.8515

TABLE S4. Computed $\hat{H}^{(2B)}_{\rm bend}$ eigenstates for the A^-_{1g} and A^-_{1u} irreps.

	A^-_{1g}		A^-_{1u}	
N	ΔE/ cm^{-1}	BSN$_{100}$	ΔE/ cm^{-1}	BSN$_{100}$
1	89.863	0.9879	89.856	0.9879
2	151.392	0.9775	151.340	0.9768
3	154.504	0.9701	154.539	0.9705
4	243.834	0.9702	243.778	0.9701
5	310.668	0.9578	310.763	0.9562
6	324.409	0.9538	324.125	0.9524
7	334.797	0.9451	334.948	0.9459
8	368.339	0.9399	368.259	0.9400
9	386.312	0.9335	385.205	0.9336
10	400.313	0.9176	400.390	0.9187
11	409.826	0.9452	409.544	0.9440
12	423.054	0.9385	424.267	0.9407
13	432.069	0.9210	431.292	0.9197
14	440.721	0.9332	440.733	0.9345
15	455.801	0.9312	454.239	0.9317
16	457.024	0.9282	456.970	0.9293
17	501.052	0.9048	501.086	0.9050
18	505.238	0.9059	504.264	0.9045
19	515.394	0.9033	512.144	0.9049
20	523.722	0.8930	523.251	0.8938
21	536.174	0.8872	536.531	0.8874
22	550.605	0.8948	552.667	0.8982
23	553.839	0.8841	553.387	0.8784
24	558.465	0.9008	560.622	0.9003
25	566.240	0.8846	566.081	0.8855
26	575.271	0.9000	571.350	0.8947
27	579.475	0.9041	575.405	0.9018
28	586.580	0.8678	582.075	0.8839
29	589.529	0.8674	589.410	0.8699
30	590.918	0.8628	591.878	0.8590

TABLE S5. Computed $\hat{H}_{\text{bend}}^{(2B)}$ eigenstates for the T_g^+ and T_u^+ irreps.

	T_g^+		T_u^+	
N	ΔE/ cm^{-1}	BSN$_{100}$	ΔE/ cm^{-1}	BSN$_{100}$
1	0.009	0.9788	0.004	0.9786
2	68.334	0.9674	68.334	0.9663
3	68.340	0.9663	68.340	0.9673
4	145.483	0.9581	145.484	0.9580
5	150.528	0.9563	150.499	0.9574
6	150.584	0.9579	150.555	0.9565
7	207.684	0.9521	207.681	0.9522
8	229.580	0.9306	229.575	0.9313
9	229.622	0.9309	229.617	0.9301
10	271.628	0.9192	271.570	0.9190
11	276.132	0.9158	276.080	0.9171
12	276.223	0.9164	276.171	0.9156
13	277.125	0.9218	277.104	0.9218
14	306.863	0.9188	306.848	0.9198
15	306.899	0.9191	306.882	0.9193
16	311.424	0.9131	311.287	0.9136
17	319.120	0.9247	319.130	0.9255
18	319.199	0.9231	319.221	0.9236
19	320.453	0.9168	320.507	0.9166
20	344.902	0.9141	344.901	0.9144
21	345.914	0.9021	345.873	0.9006
22	346.051	0.9009	346.014	0.9011
23	364.046	0.9127	364.068	0.9128
24	364.125	0.9128	364.148	0.9111
25	378.440	0.8904	378.248	0.8916
26	378.931	0.8987	378.817	0.8981
27	379.057	0.8958	379.033	0.8961
28	398.256	0.9024	397.744	0.8994
29	399.309	0.8986	398.808	0.9026
30	406.233	0.8779	406.230	0.8777
31	419.345	0.8626	419.379	0.8639
32	422.714	0.8790	422.525	0.8811
33	423.292	0.8810	423.058	0.8784
34	426.586	0.8665	426.476	0.8659
35	440.821	0.8756	440.848	0.8723
36	440.948	0.8718	440.976	0.8739
37	444.187	0.8484	444.187	0.8484
38	447.634	0.8943	447.346	0.8949
39	454.828	0.8547	454.799	0.8554
40	455.126	0.8578	455.107	0.8547

TABLE S6. Computed $\hat{H}_{\mathrm{bend}}^{(2B)}$ eigenstates for the T_g^+ and T_u^+ irreps (continued).

	T_g^+		T_u^+	
N	ΔE/ cm^{-1}	BSN$_{100}$	ΔE/ cm^{-1}	BSN$_{100}$
41	461.199	0.8407	461.055	0.8446
42	461.494	0.8450	461.323	0.8447
43	462.962	0.8658	462.909	0.8662
44	474.590	0.8466	474.633	0.8464
45	474.668	0.8486	474.718	0.8462
46	476.629	0.8925	476.623	0.8931
47	489.749	0.7953	490.024	0.8471
48	490.338	0.8505	490.121	0.8012
49	491.298	0.8467	490.899	0.8510
50	493.417	0.8463	493.451	0.8462
51	493.858	0.8495	493.791	0.8448
52	494.781	0.8210	494.558	0.8275
53	508.946	0.8330	509.204	0.8345
54	509.775	0.8282	510.038	0.8270
55	510.776	0.8133	510.363	0.8183
56	517.193	0.8050	517.227	0.8098
57	518.285	0.8414	518.223	0.8341
58	518.685	0.8304	518.511	0.8343
59	523.216	0.8052	523.069	0.8003
60	523.582	0.8138	523.315	0.8153
61	524.722	0.8013	523.977	0.8136
62	529.930	0.8328	530.225	0.8286
63	535.087	0.8014	533.509	0.7949
64	537.765	0.7956	536.405	0.8069
65	541.357	0.8041	540.535	0.7860
66	541.500	0.7832	541.446	0.8039
67	541.954	0.8080	542.059	0.8055
68	542.990	0.7717	543.206	0.7673
69	547.839	0.7903	547.561	0.7851
70	548.894	0.8019	548.240	0.7899
71	549.314	0.7814	549.120	0.7909
72	552.005	0.7971	551.758	0.7974
73	552.051	0.7894	552.355	0.8051
74	554.540	0.7953	553.493	0.7939
75	558.693	0.7933	557.733	0.8065
76	559.548	0.7874	559.285	0.7923
77	561.933	0.7329	561.785	0.7386
78	565.871	0.7999	565.252	0.7903
79	566.661	0.7838	566.593	0.7805
80	570.808	0.7768	570.667	0.7753

TABLE S7. Computed $\hat{H}^{(2B)}_{\mathrm{bend}}$ eigenstates for the T_g^- and T_u^- irreps.

	T_g^-		T_u^-	
N	$\Delta E/\ \mathrm{cm}^{-1}$	BSN_{100}	$\Delta E/\ \mathrm{cm}^{-1}$	BSN_{100}
1	24.006	0.9725	24.007	0.9715
2	24.010	0.9718	24.011	0.9727
3	89.858	0.9715	89.860	0.9714
4	151.357	0.9546	151.375	0.9544
5	154.528	0.9369	154.516	0.9366
6	193.670	0.9466	193.669	0.9467
7	193.711	0.9469	193.709	0.9466
8	215.276	0.9366	215.267	0.9358
9	215.304	0.9358	215.295	0.9363
10	243.796	0.9390	243.815	0.9386
11	270.933	0.9348	270.990	0.9346
12	271.094	0.9345	271.150	0.9346
13	301.329	0.9270	301.474	0.9276
14	301.654	0.9274	301.805	0.9277
15	310.731	0.9163	310.699	0.9171
16	322.636	0.9040	322.601	0.9038
17	322.715	0.9036	322.680	0.9032
18	324.222	0.9201	324.317	0.9204
19	334.898	0.9103	334.847	0.9105
20	344.151	0.9042	344.119	0.9035
21	344.300	0.9023	344.268	0.9036
22	368.287	0.8995	368.313	0.8991
23	376.274	0.8940	376.247	0.8940
24	376.432	0.8946	376.404	0.8938
25	382.999	0.8991	383.128	0.9036
26	383.331	0.8997	383.574	0.9004
27	385.642	0.8842	385.997	0.8831
28	400.365	0.8596	400.339	0.8590
29	409.637	0.9008	409.731	0.9007
30	414.604	0.8753	414.561	0.8760
31	414.660	0.8752	414.605	0.8719
32	422.869	0.8747	422.845	0.8757
33	423.018	0.8791	422.873	0.8793
34	423.922	0.8855	423.550	0.8826
35	431.501	0.8595	431.759	0.8589
36	434.590	0.8807	434.651	0.8787
37	434.925	0.8794	434.962	0.8794
38	440.730	0.8677	440.725	0.8685
39	444.938	0.8734	444.951	0.8742
40	445.279	0.8729	445.300	0.8748

TABLE S8. Computed $\hat{H}_{\text{bend}}^{(2B)}$ eigenstates for the T_g^- and T_u^- irreps (continued).

	T_g^-		T_u^-	
N	$\Delta E/\ \text{cm}^{-1}$	BSN_{100}	$\Delta E/\ \text{cm}^{-1}$	BSN_{100}
41	454.571	0.8791	454.834	0.8653
42	455.371	0.8495	455.843	0.8599
43	456.330	0.8511	456.244	0.8456
44	456.987	0.8713	457.001	0.8714
45	473.489	0.8349	473.616	0.8334
46	473.766	0.8348	473.850	0.8323
47	479.448	0.8661	479.538	0.8605
48	480.015	0.8535	480.044	0.8590
49	488.949	0.8493	489.271	0.8579
50	489.807	0.8580	490.095	0.8601
51	495.797	0.8481	495.967	0.8490
52	496.103	0.8495	496.326	0.8474
53	501.075	0.8282	501.063	0.8259
54	504.454	0.8236	504.713	0.8253
55	506.451	0.8394	507.559	0.8279
56	508.729	0.8318	510.469	0.8403
57	513.727	0.8029	514.621	0.7974
58	520.474	0.8087	520.898	0.8106
59	521.926	0.7957	522.434	0.8052
60	523.528	0.8031	523.634	0.8011
61	530.445	0.7965	530.419	0.7977
62	530.482	0.7983	530.492	0.7962
63	536.454	0.7780	536.337	0.7779
64	544.272	0.7842	544.564	0.7873
65	545.067	0.7856	545.316	0.7948
66	548.887	0.7596	548.800	0.7633
67	549.303	0.7728	549.032	0.7724
68	551.867	0.8029	551.343	0.8017
69	552.228	0.8089	552.020	0.7935
70	552.532	0.7912	552.950	0.7858
71	553.638	0.7743	553.748	0.7837
72	559.435	0.7840	558.927	0.7892
73	560.985	0.7917	561.007	0.7886
74	561.964	0.7797	562.479	0.7820
75	566.132	0.7509	566.184	0.7537
76	568.143	0.7687	568.298	0.7685
77	568.949	0.7672	570.373	0.7657
78	574.857	0.7649	575.256	0.7767
79	575.548	0.7824	577.026	0.7737
80	579.531	0.7629	580.431	0.7687

TABLE S9. Computed $\hat{H}_{\text{bend}}^{(2B)}$ energies for the A_{1g}^{+} irrep for three different primitive basis-set sizes: 500,000, 864,000, and 1,372,000 functions.

	500,000	864,000	1,372,000
N	E/ cm^{-1}	E/ cm^{-1}	E/ cm^{-1}
1	−4012.162	−4012.217	−4012.255
2	−3866.676	−3866.759	−3866.818
3	−3804.485	−3804.571	−3804.627
4	−3740.650	−3740.839	−3740.950
5	−3735.079	−3735.246	−3735.344
6	−3701.011	−3701.292	−3701.420
7	−3691.609	−3691.772	−3691.868
8	−3667.261	−3667.504	−3667.639
9	−3634.057	−3634.361	−3634.533
10	−3605.937	−3606.339	−3606.559
11	−3592.751	−3593.048	−3593.224
12	−3585.810	−3586.191	−3586.444
13	−3567.986	−3568.354	−3568.607
14	−3565.095	−3565.380	−3565.536
15	−3549.322	−3549.632	−3549.875
16	−3535.547	−3536.134	−3536.454
17	−3521.665	−3522.189	−3522.584
18	−3518.007	−3518.410	−3518.685
19	−3502.471	−3502.935	−3503.211
20	−3494.997	−3495.460	−3495.736
21	−3489.867	−3490.203	−3490.476
22	−3481.315	−3481.682	−3481.897
23	−3473.082	−3473.560	−3473.885
24	−3468.701	−3469.311	−3469.691
25	−3463.808	−3464.316	−3464.680
26	−3459.775	−3460.400	−3460.749
27	−3450.431	−3451.252	−3451.742
28	−3441.599	−3442.186	−3442.537
29	−3431.090	−3431.676	−3432.027
30	−3423.853	−3424.445	−3424.812
31	−3416.930	−3417.531	−3417.876
32	−3410.851	−3411.585	−3412.016
33	−3400.910	−3401.480	−3401.848
34	−3391.194	−3391.934	−3392.403
35	−3389.960	−3390.626	−3391.082
36	−3382.826	−3383.753	−3384.254
37	−3379.838	−3380.477	−3380.865
38	−3372.105	−3372.884	−3373.426
39	−3362.856	−3363.582	−3364.067
40	−3358.296	−3359.034	−3359.566

S9. SUMMARY OF RESULTS FROM THE DIAGONALIZATION OF $\hat{H}_{\text{bend}}$

Following are results pertaining to the 30/80 lowest-energy $\hat{H}_{\text{bend}}$ eigenstates computed for each of the A/T G_{48} irreps. In each Table ΔE is the energy of the state relative to that of the $\hat{H}_{\text{bend}}$ ground state ($A_{1g}^{+}(1)$) at -3968.752 cm^{-1}. BSN$_1$ is the basis-state norm corresponding to the single largest contributing basis state (i.e., $\hat{H}_{\text{bend}}^{(2B)}$ eigenstate) to each $\hat{H}_{\text{bend}}$ eigenstate.

TABLE S10. Computed $\hat{H}_{\text{bend}}$ eigenstates for the A_{1g}^{+} and A_{1u}^{+} irreps.

	A_{1g}^{+}		A_{1u}^{+}	
N	ΔE/ cm^{-1}	BSN$_1$	ΔE/ cm^{-1}	BSN$_1$
1	0.000	0.9980	0.010	0.9980
2	146.608	0.9976	146.604	0.9976
3	207.029	0.9974	207.035	0.9974
4	274.256	0.9902	274.361	0.9908
5	277.493	0.9935	277.529	0.9938
6	316.042	0.9373	316.345	0.9342
7	327.830	0.9386	327.767	0.9353
8	350.156	0.9818	350.150	0.9818
9	384.067	0.9886	384.675	0.9890
10	415.631	0.9286	415.704	0.9294
11	421.605	0.9937	421.524	0.9942
12	432.061	0.9257	432.340	0.9265
13	448.952	0.6586	449.036	0.5347
14	458.286	0.6745	458.887	0.5469
15	472.419	0.9721	472.609	0.9746
16	490.985	0.9486	491.036	0.9496
17	500.356	0.7222	500.050	0.7217
18	501.583	0.7739	501.258	0.6970
19	519.312	0.9333	520.481	0.9455
20	526.582	0.9706	526.716	0.9377
21	533.645	0.9151	534.605	0.9142
22	538.909	0.9203	539.148	0.8706
23	551.397	0.7349	552.319	0.4685
24	553.604	0.7378	553.789	0.5149
25	561.235	0.8478	562.206	0.8173
26	565.401	0.7860	567.932	0.5768
27	571.096	0.7709	574.914	0.6178
28	582.872	0.8791	583.594	0.8859
29	590.225	0.9469	590.375	0.9472
30	602.160	0.9315	601.794	0.9389

TABLE S11. Computed $\hat{H}_{\text{bend}}$ eigenstates for the $A^{-}_{2g/3g}$ and $A^{-}_{2u/3u}$ irreps.

	$A^{-}_{2g/3g}$		$A^{-}_{2u/3u}$	
N	ΔE/ cm^{-1}	BSN$_1$	ΔE/ cm^{-1}	BSN$_1$
1	23.144	0.9979	23.144	0.9979
2	193.401	0.9962	193.407	0.9962
3	216.293	0.9968	216.316	0.9968
4	273.917	0.9897	273.793	0.9895
5	311.012	0.9796	310.638	0.9801
6	324.557	0.9802	324.631	0.9810
7	350.039	0.9802	350.128	0.9801
8	381.149	0.9226	381.227	0.9153
9	394.483	0.9170	394.083	0.9103
10	420.793	0.9487	420.833	0.9511
11	427.398	0.9579	427.582	0.9608
12	444.164	0.8855	444.345	0.8800
13	453.983	0.8754	453.718	0.8667
14	464.622	0.9726	464.494	0.9709
15	485.059	0.9532	484.612	0.9502
16	491.002	0.9525	491.005	0.9461
17	498.810	0.8687	498.512	0.8633
18	506.742	0.8913	506.429	0.8880
19	522.881	0.8930	519.957	0.9179
20	531.622	0.7893	530.051	0.8248
21	540.456	0.8666	540.113	0.8705
22	556.258	0.9431	555.936	0.9272
23	561.065	0.8267	561.285	0.8181
24	564.410	0.8186	564.345	0.8050
25	574.885	0.9442	573.966	0.9235
26	583.433	0.8971	581.700	0.9062
27	594.866	0.8205	593.109	0.8436
28	601.487	0.7892	601.326	0.8722
29	603.816	0.6469	606.444	0.6489
30	609.790	0.6991	612.450	0.6580

TABLE S12. Computed $\hat{H}_{\mathrm{bend}}$ eigenstates for the $A^+_{2g/3g}$ and $A^+_{2u/3u}$ irreps.

	$A^+_{2g/3g}$		$A^+_{2u/3u}$	
N	ΔE/ cm^{-1}	BSN$_1$	ΔE/ cm^{-1}	BSN$_1$
1	66.161	0.9975	66.166	0.9975
2	151.488	0.9968	151.555	0.9969
3	229.533	0.9964	229.548	0.9964
4	280.136	0.9753	280.244	0.9753
5	314.225	0.9537	314.288	0.9530
6	321.195	0.9787	321.175	0.9779
7	350.164	0.9855	350.251	0.9852
8	372.141	0.9723	372.067	0.9734
9	389.821	0.9573	389.824	0.9618
10	407.878	0.9542	409.036	0.9566
11	431.655	0.9740	432.311	0.9735
12	449.673	0.9683	449.617	0.9671
13	462.172	0.9132	462.146	0.9286
14	468.846	0.9063	469.087	0.9224
15	482.916	0.9482	483.045	0.9488
16	501.595	0.6195	501.597	0.6377
17	503.058	0.6329	503.930	0.6507
18	523.287	0.8476	522.488	0.9440
19	524.946	0.8344	524.988	0.9254
20	532.585	0.8942	532.370	0.9133
21	545.560	0.7827	547.512	0.5700
22	550.650	0.8438	550.983	0.5892
23	561.182	0.9251	561.720	0.8609
24	563.961	0.9001	564.513	0.8187
25	570.139	0.9517	571.789	0.9401
26	579.467	0.8456	579.655	0.8022
27	585.758	0.8460	587.122	0.8227
28	593.866	0.8887	595.405	0.7688
29	597.875	0.8950	600.245	0.7731
30	610.184	0.9414	613.218	0.5730

TABLE S13. Computed $\hat{H}_{\text{bend}}$ eigenstates for the A_{1g}^{-} and A_{1u}^{-} irreps.

	A_{1g}^{-}		A_{1u}^{-}	
N	ΔE/ cm^{-1}	BSN_1	ΔE/ cm^{-1}	BSN_1
1	87.370	0.9966	87.372	0.9965
2	152.100	0.9953	152.060	0.9953
3	154.334	0.9969	154.363	0.9970
4	244.902	0.9964	244.859	0.9964
5	317.981	0.8929	318.068	0.8880
6	328.144	0.8457	327.979	0.8419
7	341.777	0.9333	341.846	0.9339
8	371.173	0.9774	371.133	0.9761
9	391.940	0.9794	391.193	0.9783
10	407.176	0.9657	407.196	0.9688
11	412.353	0.9842	412.160	0.9857
12	434.533	0.8914	435.810	0.8384
13	441.790	0.7995	441.001	0.7728
14	446.652	0.8921	446.803	0.9190
15	464.998	0.7525	464.624	0.6530
16	466.853	0.7454	465.487	0.6767
17	511.806	0.9576	511.465	0.9156
18	514.698	0.9804	514.096	0.9449
19	526.029	0.9341	524.204	0.9423
20	533.839	0.9682	533.201	0.9695
21	548.333	0.9787	548.415	0.9769
22	563.039	0.6830	563.009	0.8587
23	564.892	0.6432	565.999	0.8333
24	567.971	0.8573	569.695	0.8072
25	579.117	0.7800	579.028	0.7556
26	585.849	0.5478	585.553	0.5491
27	594.551	0.5242	590.921	0.7606
28	599.455	0.3521	595.063	0.7146
29	601.602	0.5848	601.251	0.4946
30	605.958	0.5344	603.653	0.4527

TABLE S14. Computed $\hat{H}_{\text{bend}}$ eigenstates for the T_g^+ and T_u^+ irreps.

	T_g^+		T_u^+	
N	$\Delta E/$ cm^{-1}	BSN$_1$	$\Delta E/$ cm^{-1}	BSN$_1$
1	0.070	0.9981	0.066	0.9981
2	66.258	0.9879	66.258	0.9549
3	66.264	0.9879	66.263	0.9549
4	146.709	0.9977	146.709	0.9977
5	151.632	0.9960	151.611	0.9968
6	151.672	0.9960	151.650	0.9968
7	207.183	0.9976	207.181	0.9976
8	229.698	0.9952	229.693	0.9958
9	229.734	0.9952	229.728	0.9958
10	274.515	0.9908	274.480	0.9906
11	277.734	0.9919	277.722	0.9917
12	280.368	0.9731	280.327	0.9756
13	280.430	0.9754	280.391	0.9733
14	314.419	0.9420	314.401	0.9379
15	314.467	0.9420	314.442	0.9375
16	316.465	0.9356	316.367	0.9365
17	321.387	0.9784	321.392	0.9765
18	321.465	0.9743	321.472	0.9789
19	328.003	0.9333	328.024	0.9351
20	350.385	0.7044	350.385	0.6242
21	350.447	0.7135	350.414	0.5797
22	350.556	0.9779	350.531	0.9322
23	372.335	0.9396	372.358	0.9414
24	372.400	0.9388	372.428	0.9407
25	384.750	0.6962	384.548	0.8629
26	390.069	0.5537	390.059	0.4907
27	390.172	0.4058	390.174	0.4686
28	408.588	0.9368	408.200	0.9352
29	409.360	0.9374	408.986	0.9357
30	416.016	0.9281	415.996	0.9279
31	421.857	0.9945	421.884	0.9942
32	432.087	0.8396	431.950	0.9399
33	432.659	0.7220	432.301	0.5539
34	432.732	0.8478	432.698	0.5332
35	449.384	0.5646	449.362	0.6050
36	449.949	0.9580	449.966	0.9465
37	450.069	0.9535	450.091	0.9518
38	459.050	0.5803	458.856	0.6230
39	462.469	0.9180	462.476	0.9152
40	462.591	0.9227	462.601	0.9195

TABLE S15. Computed $\hat{H}_{\text{bend}}$ eigenstates for the T_g^+ and T_u^+ irreps (continued).

	T_g^+		T_u^+	
N	$\Delta E/\ \text{cm}^{-1}$	BSN_1	$\Delta E/\ \text{cm}^{-1}$	BSN_1
41	469.291	0.9057	469.213	0.9022
42	469.468	0.9148	469.386	0.9065
43	472.964	0.9722	472.906	0.9705
44	483.331	0.5854	483.279	0.6088
45	483.438	0.5855	483.390	0.6095
46	491.465	0.9504	491.453	0.9499
47	500.603	0.5704	500.708	0.4705
48	501.651	0.4566	501.622	0.3210
49	501.846	0.6178	502.000	0.4614
50	502.346	0.6493	502.325	0.6202
51	503.608	0.6913	503.372	0.5773
52	504.399	0.6487	504.048	0.6900
53	520.576	0.8838	520.184	0.6832
54	522.829	0.9383	523.104	0.8778
55	523.553	0.8485	523.809	0.6312
56	525.248	0.8748	525.224	0.7747
57	525.480	0.9080	525.471	0.7772
58	527.162	0.9229	527.120	0.9201
59	532.788	0.7882	532.844	0.8636
60	532.930	0.8512	533.021	0.4478
61	534.872	0.8299	534.533	0.4715
62	539.249	0.8770	539.204	0.8939
63	546.645	0.7307	545.933	0.7844
64	547.966	0.5182	547.360	0.6276
65	551.035	0.3465	550.838	0.5838
66	551.470	0.3644	551.350	0.7635
67	552.963	0.9051	552.722	0.4608
68	553.886	0.8215	553.848	0.4639
69	561.627	0.8629	561.525	0.8564
70	562.217	0.7861	561.774	0.7912
71	562.482	0.7115	562.328	0.7722
72	564.486	0.6321	564.450	0.6810
73	564.936	0.5923	564.573	0.6376
74	567.719	0.6438	566.867	0.7186
75	570.642	0.7169	570.529	0.9349
76	572.410	0.9255	571.001	0.5506
77	573.942	0.5817	573.247	0.4647
78	579.929	0.7837	579.732	0.7992
79	580.305	0.7732	580.284	0.7543
80	583.717	0.8826	583.504	0.8824

TABLE S16. Computed $\hat{H}_{\text{bend}}$ eigenstates for the T_g^- and T_u^- irreps.

	T_g^-		T_u^-	
N	ΔE/ cm^{-1}	BSN$_1$	ΔE/ cm^{-1}	BSN$_1$
1	23.215	0.8917	23.216	0.8250
2	23.218	0.8917	23.219	0.8250
3	87.476	0.9967	87.476	0.9967
4	152.185	0.9955	152.198	0.9955
5	154.493	0.9971	154.483	0.9971
6	193.554	0.9961	193.551	0.9958
7	193.583	0.9961	193.581	0.9959
8	216.447	0.9966	216.439	0.9968
9	216.470	0.9966	216.465	0.9968
10	245.047	0.9966	245.058	0.9966
11	273.962	0.9861	274.004	0.9856
12	274.079	0.9862	274.124	0.9857
13	310.804	0.9784	310.929	0.9781
14	311.086	0.9783	311.213	0.9780
15	318.242	0.8930	318.211	0.8947
16	324.832	0.9766	324.808	0.9788
17	324.882	0.9765	324.858	0.9788
18	328.274	0.8475	328.326	0.8488
19	342.058	0.9352	342.034	0.9350
20	350.289	0.9801	350.257	0.9789
21	350.407	0.9800	350.373	0.9789
22	371.452	0.9772	371.466	0.9776
23	381.438	0.9108	381.407	0.9138
24	381.548	0.9054	381.520	0.9082
25	391.701	0.9143	391.948	0.9237
26	394.389	0.8874	394.550	0.8317
27	394.706	0.8300	394.804	0.8892
28	407.505	0.9684	407.499	0.9673
29	412.558	0.9857	412.619	0.9852
30	421.145	0.5744	421.133	0.5033
31	421.179	0.5746	421.166	0.5025
32	427.804	0.9148	427.750	0.8757
33	427.909	0.9510	427.845	0.7862
34	435.712	0.8269	435.295	0.7868
35	441.589	0.7835	441.850	0.7934
36	444.549	0.8802	444.489	0.8771
37	444.793	0.8793	444.738	0.8765
38	447.107	0.9109	447.055	0.9023
39	454.066	0.8449	454.154	0.8480
40	454.301	0.8464	454.397	0.8489

TABLE S17. Computed $\hat{H}_{\text{bend}}$ eigenstates for the T_g^- and T_u^- irreps (continued).

	T_g^-		T_u^-	
N	ΔE/ cm^{-1}	BSN$_1$	ΔE/ cm^{-1}	BSN$_1$
41	464.645	0.9642	464.589	0.6318
42	464.941	0.5332	465.238	0.9578
43	465.386	0.4761	465.376	0.6994
44	466.306	0.3995	466.738	0.4092
45	485.002	0.9451	485.177	0.9446
46	485.402	0.9446	485.534	0.9443
47	491.352	0.8913	491.365	0.9002
48	491.552	0.8888	491.522	0.8974
49	498.857	0.8546	498.952	0.8593
50	499.211	0.8672	499.314	0.8710
51	506.811	0.7068	506.909	0.7048
52	507.116	0.7263	507.236	0.7251
53	512.034	0.9291	512.151	0.9451
54	514.668	0.9422	514.851	0.9333
55	520.313	0.9093	521.153	0.8737
56	522.093	0.8877	523.333	0.8648
57	525.541	0.9134	526.040	0.9156
58	530.147	0.7081	530.634	0.7348
59	531.640	0.7230	532.259	0.7095
60	533.971	0.9451	534.153	0.9588
61	540.514	0.6788	540.611	0.7557
62	540.842	0.6659	540.974	0.7430
63	548.863	0.9780	548.837	0.9786
64	556.361	0.8732	556.477	0.8686
65	556.705	0.8859	556.806	0.8797
66	561.458	0.8043	561.409	0.8092
67	561.848	0.7554	561.740	0.7608
68	563.563	0.4764	563.598	0.5408
69	564.555	0.5653	564.564	0.7686
70	565.000	0.7990	564.980	0.7200
71	566.249	0.5503	565.908	0.5632
72	569.311	0.7437	568.763	0.8339
73	574.255	0.8292	574.479	0.8874
74	575.211	0.8927	575.592	0.9253
75	579.494	0.7604	579.519	0.7705
76	582.043	0.9037	582.371	0.8933
77	583.176	0.8607	584.120	0.8800
78	586.191	0.7287	586.256	0.4687
79	592.614	0.5762	592.782	0.4665
80	593.225	0.6066	594.995	0.4297

REFERENCES

[1]V. A. Mandelshtam and H. S. Taylor, J. Chem. Phys. **106**, 5085 (1997).

[2]M. R. Wall and D. Neuhauser, J. Chem. Phys. **102**, 8011 (1995).
